# Circulating microRNA profile of long‐lived Okinawans identifies novel potential targets for optimizing lifespan and health span

**DOI:** 10.1111/acel.14191

**Published:** 2024-05-15

**Authors:** Sarah Noureddine, Augusto Schneider, Sydney Strader, Xiang Zhu, Joseph Dhahbi, Richard Allsopp, D. Craig Willcox, Timothy A. Donlon, Michio Shimabukuro, Moritake Higa, Makoto Suzuki, Trevor Torigoe, Sarah Ashiqueali, Hariom Yadav, Bradley J. Willcox, Michal M. Masternak

**Affiliations:** ^1^ University of Central Florida College of Medicine, Burnett School of Biomedical Sciences Orlando Florida USA; ^2^ Faculdade de Nutrição Universidade Federal de Pelotas Pelotas Brazil; ^3^ Department of Medical Education, School of Medicine California University of Science & Medicine Colton California USA; ^4^ Institute for Biogenesis Research, John A. Burns School of Medicine University of Hawai'i Honolulu Hawaii USA; ^5^ Center of Biomedical Research Excellence for Translational Research on Aging, Kuakini Medical Center Honolulu Hawaii USA; ^6^ Okinawa Research Center for Longevity Science Urasoe Japan; ^7^ Department of Human Welfare Okinawa International University Ginowan Japan; ^8^ Department of Cell and Molecular Biology, John A. Burns School of Medicine University of Hawai'i Honolulu Hawaii USA; ^9^ Department of Diabetes, Endocrinology and Metabolism Fukushima Medical University, School of Medicine Fukushima Japan; ^10^ Diabetes and Life‐Style Related Disease Center, Tomishiro Central Hospital Tomishiro Japan; ^11^ USF Center for Microbiome Research Microbiomes Institute, University of South Florida Morsani College of Medicine Tampa Florida USA; ^12^ Department of Geriatric Medicine, John A. Burns School of Medicine University of Hawai'i Honolulu Hawaii USA; ^13^ Department of Head and Neck Surgery Poznan University of Medical Sciences Poznan Poland

**Keywords:** aging, FOXO3, longevity, microRNAs, Okinawan nonagenarians

## Abstract

Nonagenarians and centenarians serve as successful examples of aging and extended longevity, showcasing robust regulation of biological mechanisms and homeostasis. Given that human longevity is a complex field of study that navigates molecular and biological mechanisms influencing aging, we hypothesized that microRNAs, a class of small noncoding RNAs implicated in regulating gene expression at the post‐transcriptional level, are differentially regulated in the circulatory system of young, middle‐aged, and nonagenarian individuals. We sequenced circulating microRNAs in Okinawan males and females <40, 50–80, and >90 years of age accounting for *FOXO3* genetic variations of single nucleotide polymorphism (SNP) rs2802292 (TT ‐ common vs. GT ‐ longevity) and validated the findings through RT‐qPCR. We report five microRNAs exclusively upregulated in both male and female nonagenarians with the longevity genotype, play predictive functional roles in TGF‐β, FoxO, AMPK, Pi3K‐Akt, and MAPK signaling pathways. Our findings suggest that these microRNAs upregulated in nonagenarians may provide novel insight into enhanced lifespan and health span. This discovery warrants further exploration into their roles in human aging and longevity.

## Graphical abstract

Centenarians and nonagenarians demonstrate successful aging, a phenomenon described as aging devoid of advanced disease, impaired cognitive function and reduced physical ability. Given the potential for miRNAs to be used as biomarkers of aging or anti‐aging therapies, we identified five differentially expressed circulating miRNAs in Okinawan nonagenarians involved in key age‐related pathways.

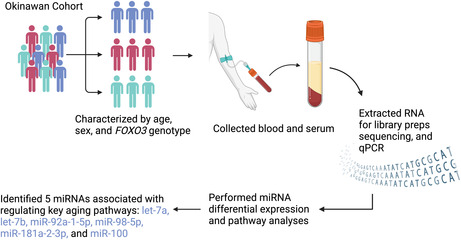

**Abbreviations**
AKT: protein kinase B
AMPK: Adenosine monophosphate‐activated protein kinase
cAMP: cyclic adenosine monophosphate
CDKN1A: cyclin dependent kinase inhibitor 1A
cDNA: complimentary deoxyribonucleic acid
CT: cycle threshold
ELK4: ETS domain‐containing protein 4
ErbB: erythroblastic leukemia viral oncogene homologue
FoxO: forkhead box transcription factor
*FOXO3*
: Forkhead box 03 gene
Homer1: Homer scaffold protein 1
IGF‐1: insulin‐like growth factor 1
IGF‐1R: IGF‐1 receptor
IRS: Insulin receptor substrate
MAPK: Mitogen‐activated protein kinase
miRNA: microRNA
mRNA: messenger RNA
mTOR: mammalian target of rapamycin
p53: tumor protein p53
Pi3K: phosphoinositide 3‐kinase
PPP1R15 β: protein phosphatase 1 regulatory subunit 15 beta
RNA: ribonucleic acid
ROS: reactive oxygen species
RT‐qPCR: real time quantitative polymerase chain reaction
SASP: senescence associated secretory phenotype
SnC: senescent cell
SNP: single nucleotide polymorphism
SOS2: son of sevenless 2
SRE: serum response element
SRF: serum response factor
sRNA: small RNA
TGF‐β R: TGF‐β receptor
TGF‐β: transforming growth factor beta
UTR: untranslated region

## 1 INTRODUCTION

Centenarians and nonagenarians demonstrate successful aging, a phenomenon described as aging devoid of advanced disease, impaired cognitive function and reduced physical ability (Engberg et al., 2009). In addition to exhibiting improved health span, centenarians surpass the average life expectancy by over two decades (Engberg et al., 2009). Across the globe, Okinawa ranks among the highest for both life expectancy and prevalence of centenarians, with a frequency of 6 centenarians per 10,000 individuals (Buchholz, 2021; Willcox, Willcox, et al., 2008). Long‐lived Okinawans also demonstrate reduced signs of age‐associated diseases, eliciting interest in identifying genetic and environmental contributors to the population's overall improved health and longevity (Bernstein et al., 2004).

Furthermore, Okinawan centenarians and long‐lived nonagenarians exhibit advanced regulation of biological mechanisms and homeostasis, and more specifically, display greater insulin sensitivity. Their improved insulin sensitivity has been somewhat attributed to variations of the forkhead box 03 (*FOXO3*) gene (Willcox, Donlon, et al., 2008). The *FOXO3* gene encodes a key regulator of the insulin‐IGF‐1 signaling pathway, central to metabolic homeostasis and notably deregulated with age (López‐Otín et al., 2013). A single‐nucleotide polymorphism (SNP) in the *FOXO3* gene was previously identified to be linked with longevity in Okinawan centenarians and nonagenarians. Carriers of the G allele are associated with improved health at baseline (Willcox, Donlon, et al., 2008). These findings were supported by a separate study investigating *FOXO3* SNPs in German centenarians and nonagenarians that concluded *FOXO3* is also associated with human longevity (Flachsbart et al., 2009; Frankum et al., 2022).

In addition to evaluating the role of *FOXO3* in longevity, there is growing support for the role of metabolic pathways—including Insulin‐IGF‐1 signaling—in aging. Thus, investigating the molecular and genetic factors that modulate metabolic processes could provide insight into the mechanisms that support extended lifespan in nonagenarians. A class of small noncoding RNAs known as microRNAs (miRNAs) repress the translation of sequence‐compatible messenger RNAs (mRNAs) through destabilization or degradation of the target mRNA and/or through 3′UTR (untranslated region) binding (Nunes et al., 2022). Therefore, several pathways can be regulated through this mechanism, including longevity (Marchegiani et al., 2023). Several miRNAs have already been proposed to promote longevity in humans, including miRs‐186, −29, −18a, − 434‐3p, and −let‐7 (Kinser & Pincus, 2019). Other studies have also established uniquely over‐expressed miRNAs in Spanish centenarians compared to octogenarians and young individuals comprising miRs‐21, −130a, and −494, that appear to be involved in regulating cell death, cardiac development, and apoptosis, respectively (Serna et al., 2012). Given the potential for miRNAs to be used as biomarkers of aging or anti‐aging therapies (Jueraitetibaike et al., 2023), we were interested in identifying differentially expressed circulating miRNAs and their pathway interactions in Okinawan nonagenarians. To address this, we sequenced, and validated through qPCR, circulating miRNAs in Okinawans <40, 50–80, and ≥90 years of age accounting for differences in age, sex, and *FOXO3* rs2802292 genotype (TT and TG).

## 2 MATERIALS AND METHODS

### 2.1 Participant inclusion and serum collection

**FIGURE 1:**
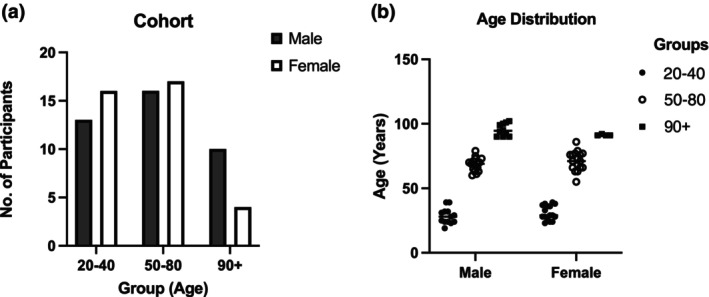
(a) Number of male and female participants per age group. (b) Age distribution of males and females per age group.

Male and female participants (*n* = 320) ranging in age from 19 to 104 years were recruited during annual health examinations, following written informed consent, through Tomishiro Central Hospital (Tomishiro City, Okinawa, Japan) and affiliated clinics and facilities throughout the Okinawa prefecture. Subjects were recruited during nationally required annual health screening examinations, thereby mitigating potential bias toward healthier participants. Recruitment occurred between May 2018 and July 2019. Principal inclusion criteria focused on healthy individuals or individuals with non‐severe medical conditions over the age of 18 years. Subjects were excluded from participation if they were (a) aged <18 years, (b) had a severe medical condition, such as diabetes mellitus with glycated hemoglobin A1c ≥ 8.5%; recent (within a year) onset of ischemic heart disease, cerebrovascular disease, and cancer, (c) were taking anti‐diabetic, lipid‐lowering or anti‐hypertensive medications within 18 h prior to the blood sampling that potentially affect microRNA measurements, (d) exhibited severe dementia or an inability to comprehend the informed consent, (e) had a known genetic disease or disability, or (f) were restricted from participation by the subject's attending physician. Demographic characteristics of participants are shown in Table S1. This study was conducted following approval by the Ethics Committees from Tomishiro Central Hospital (H25R008), Fukushima Medical University (#30167). Hence, based on the inclusion–exclusion criteria, *n* = 76 participants were included in this study. A breakdown of number of participants and age distribution is presented in Figure 1.

To isolate serum from included participants, 2 mL of Dulbecco's phosphate buffered saline (DPBS) with calcium and magnesium (ThermoFisher Scientific, Waltham, Massachusetts, USA) were added to 2 mL of whole blood and mixed at room temperature. Three milliliter of Ficoll‐Paque PREMIUM (GE Healthcare/Cytiva, Chicago, Illinois, USA) were added to a new 10 mL centrifuge tube. Four milliliter of the diluted blood sample was carefully layered on top of the Ficoll‐Paque Premium, ensuring that there was no mixing between the layers, before being centrifuged at 400× *g* for 40 min with the brake turned off. After centrifugation, the upper layer containing the plasma was removed.

### 2.2 RNA isolation

200 μL of serum were homogenized with 5 volumes of QIAzol Lysis Reagent, combined with chloroform and mixed vigorously to ensure subsequent phase separation. Samples were incubated for 3 min and centrifuged at 12,000× *g* at 4°C for 15 min. Following phase separation, RNA was extracted using the QIAGEN miRNeasy Serum/Plasma kit (Hilden, Germany). All steps were performed in accordance with the manufacturer's protocol.

### 2.3 Library Prep and small RNA sequencing

To prepare libraries for sequencing, 10.5 μL of total undiluted RNA was combined with the NEXTFLEX Small RNA Seq. kit V3 reagents as per the manufacturer's protocol (Perkin‐Elmer, Waltham, Massachusetts, USA). Samples were purified using the gel‐selection method in a 6% TBE‐PAGE gel. Gel electrophoresis was performed at 145 V for approximately 30 min. Bands were visualized on a UV transilluminator following staining with SYBR Gold (Invitrogen, Waltham, Massachusetts) in accordance with the manufacturer's instructions. Bands visualized at approximately 150 bp were excised, homogenized with disposable pestles, and incubated overnight at room temperature with agitation in the NEXTFLEX Elution Buffer (Perkin‐Elmer, Waltham, Massachusetts). Homogenized gel fragments and eluent were then transferred to Corning® Costar® Spin‐X® centrifuge tube filters (Glendale, Arizona, USA) following overnight incubation and centrifuged at 16,000× *g* for 2 min. The collected eluent was then purified using the QIAquick Gel Extraction Kit (QIAGEN, Hilden, Germany) as per the user‐developed protocol by Sambrook et al. (1989). The final libraries were then outsourced for quality check, pooling, and small RNA sequencing via Illumina HiSeq 2x150 system (Admera Health). Based on QC results, at least *n* = 4 per sex and age group was selected for pooling/sequencing. Samples were multiplexed and run on 2 separate lanes for a total of 32 libraries.

### 2.4 sRNA seq fold change and relative expression

**FIGURE 2:**
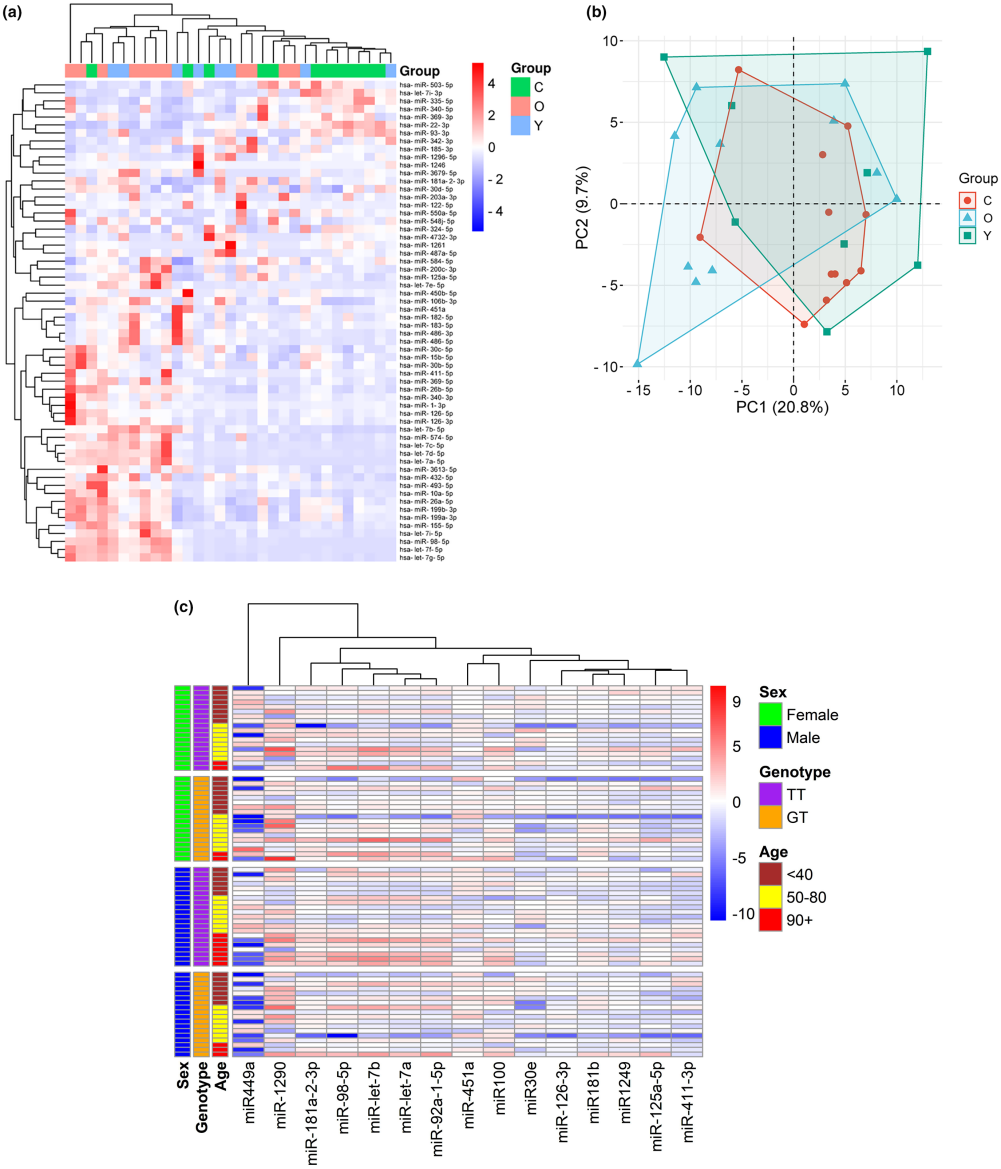
(a) Heatmap for unsupervised hierarchical clustering of differentially expressed miRNAs across study cohort. (b) Principal component analysis of miRNAs in the 3 studied groups. (c) Heatmap of post‐sequencing candidate miRNAs quantified through RT‐qPCR and their association with sex, age, and genotype.

Alignment and quantification of miRNA libraries was performed using cutadapt and bowtie2 (Langmead et al., 2019) as described previously (Rueda et al., 2015). Statistical analyses of differentially expressed miRNAs was performed using EdgeR (Robinson et al., 2010) on the R software (3.2.2). miRNAs with a *p*‐value <0.05 and FC >2.0 were considered as upregulated while miRNAs with a *p* < 0.05 and FC < 0.50 were considered as downregulated. A summary of the differentially expressed miRNAs is shown in Figure 2a. A principal component analysis was performed to determine the variations in the datasets. This analysis established a PC1 of 20.8% and a PC2 of 9.7% (Figure 2b). Furthermore, volcano plots were derived from the sequencing datasets as shown in Figure S1. Based on these analyses, a narrowed list of miRNAs differentially expressed with age and/or changes in genotype was derived for further analysis through RT‐qPCR using the full sample set of *n* = 76.

### 2.5 cDNA Prep and quantitative real time PCR

To validate differentially expressed miRNAs derived post‐small RNA‐seq using all samples from included participants (*n* = 76), cDNA was synthesized from total RNA using the Applied Biosystems™ TaqMan™ Advanced miRNA cDNA Synthesis Kit (ThermoFisher Scientific, Waltham, Massachusetts, USA) for subsequent quantitative real‐time PCR (RT‐qPCR). Each reaction step was performed using the Bio‐Rad Thermal Cycler T100. Ten microliter of the final cDNA product (50 μL) was diluted in 90 μL of RNase‐free water to narrow the range of the control miRNA‐16 cycle threshold (C_T_) values with RT‐qPCR. 2.5 μL of diluted cDNA per sample was then combined with 7.5 μL of the TaqMan Fast Advanced Master Mix and each respective TaqMan miRNA assay for a sample total of 10 μL per well. RT‐qPCR was then performed for each sample in duplicates using the QuantStudio 7 Flex Real‐Time PCR system and the MicroAmp™ Fast Optical 96‐Well Reaction Plate with Barcode, 0.1 mL and Adhesive PCR Plate Seals (ThermoFisher Scientific) to quantify expression of miRNAs‐16, −451a, −let‐7a, −let‐7b, −92a‐1‐5p, −98‐5p, −125a‐5p, −126‐3p, −181a‐2‐3p, −411‐3p, −1290, −30e, −100, −181b‐5p, −449a‐5p, −1249, respectively, using the TaqMan MicroRNA Assays (Table S2).

RT‐qPCR was normalized using miR‐16 as the housekeeping gene with a maximum range of 3 miR‐16 C_T_ values between all samples. Relative expression of each miRNA was then calculated using the −∆∆C_T_ method as described previously (Noureddine et al., 2023; Piotrowski et al., 2021).

### 2.6 Prediction of microRNA pathway interactions and gene targets

To evaluate predictive pathway associations for differentially expressed microRNAs validated through RT‐qPCR, the DIANA Tool miRPath v3 was used to generate a comprehensive list of gene and pathway targets for each microRNA using the micro‐T‐CDS v5 database (Paraskevopoulou et al., 2013; Vlachos et al., 2015). To further demonstrate miRNA‐gene interactions, MIENTURNET, a miRNA‐target enrichment and network‐based analysis tool was utilized (Licursi et al., 2019).

## 3 RESULTS

### 3.1 Age impacts expression of miRNAs in young, middle‐elderly aged, and nonagenarian individuals

To explore the impact of age on the expression profile of miRNAs in circulation, we evaluated differentially expressed miRNAs in serum isolated from young (<40 years of age), middle aged‐elderly (50–80 years of age) and nonagenarians (>90 years of age) Okinawan individuals. miR‐1246, miR‐3679‐5p, miR‐183‐5p, miR‐486‐3p, miR‐486‐5p, let‐7a‐5p, let‐7b‐5p, miR‐182‐5p, miR‐451a, and miR‐342‐3p were downregulated in young individuals compared to the nonagenarian cohort (Table S3). These miRNAs are predicted to target genes involved in TGF‐β, Pi3k‐Akt, AMPK, FoxO, mTOR, and MAPK signaling pathways, suggesting that these pathways may be upregulated in individuals <40 years of age compared to nonagenarians (Table S4). Conversely, miR‐335‐5p, miR‐369‐3p, and miR‐503‐5p were found to be upregulated in circulation of the younger cohort compared to nonagenarians (Table S3). These miRNAs demonstrate potential in targeting genes involved in signaling pathways regulating pluripotency of stem cells (Table S5).

When comparing middle aged and elderly individuals (50–80 years of age) to nonagenarians, 35 miRNAs were downregulated (Table S6). These miRNAs are predicted to have functional roles in regulating pluripotency of stem cells, FoxO, Ras, Pi3K‐Akt, MAPK, mTOR, TGF‐β, p53, and AMPK signaling pathways as well as long‐term depression, suggesting the middle‐aged and elderly group demonstrate increased expression of nutrient‐sensing pathways and onset of long‐term depression when compared to the nonagenarian cohort (Table S7). On the other hand, miR‐22‐3p, miR‐450b‐5p, miR‐93‐3p, and let‐7i‐3p were upregulated in the 50–80 age group (Table S6) and appear to target genes involved in similar pathways. However, the number of genes targeted are less than the genes targeted by the upregulated miRNAs, suggesting the overall outcome is likely upregulation of the pathways targeted by the downregulated miRNAs (Tables S7 and S8).

Furthermore, sRNA‐seq analysis comparing the <40 group to individuals 50–80 years of age revealed downregulation of 8 miRNAs and upregulation of 20 miRNAs (Table S9) in the younger cohort. Pathway analysis revealed the group of downregulated miRNAs possibly function by repressing genes involved in regulating pluripotency of stem cells and TGF‐β signaling (Table S10). On the other hand, the upregulated miRNAs demonstrate predictive function in regulating FoxO, Ras, TGF‐β, Pi3K‐Akt, MAPK, mTOR, insulin signaling, and type II diabetes mellitus (Table S11).

Together, these results indicate that miRNA expression patterns greatly differ with age and could offer insight into the regulation of pathways involved in lifespan‐extension.

### 3.2 sRNA‐sequencing reveals genotype and miRNA associations across Okinawan cohorts

Okinawan nonagenarians exhibit enhanced insulin sensitivity that has been attributed to the *FOXO3* genetic variations. Previous studies have demonstrated a correlation between the G allele and improved health (Willcox, Donlon, et al., 2008). Hence, to account for genotypic influence on circulating miRNA expression patterns, we analyzed differentially expressed miRNAs in young, middle aged‐elderly, and nonagenarian Okinawan individuals with either TT or TG alleles. miR‐92a‐1‐5p, miR‐411‐3p, miR‐1254, miR‐889‐3p, miR‐323a‐3p, miR‐379‐5p, miR‐369‐3p, miR‐487b‐3p, and miR‐335‐5p were determined to be downregulated in TG vs TT individuals while miR‐92a‐3p, miR‐320b, miR‐744‐5p, mIR‐3184‐5p, miR‐185‐3p, miR‐320a, miR‐23a‐5p, miR‐486‐3p, miR‐193a‐5p, miR‐3591‐3p, miR‐1307‐5p, miR‐122‐5p, miR‐450b‐5p, miR‐4732‐5p, and miR‐1290 were upregulated (Table 1). Predicted pathway interactions of downregulated miRNAs in TG versus TT include TGF‐β and p53 signaling, as well as pathways regulating pluripotency of stem cells (Table S12). Conversely, upregulated miRNAs in TG individuals target ErbB, FoxO, AMPK, mTOR, and cAMP signaling, as well as in long‐term depression, suggesting individuals with TG alleles have increased regulation of important nutrient sensing pathways typically deregulated with age (Table S13).

**TABLE 1:** Differentially expressed microRNAs demonstrating genotype associations in young, middle‐aged, and nonagenarians individuals (<40, 50–80, and >90 years of age, respectively). logFC indicates log fold change and logCPM represents log counts per million indicative of expression levels; *p* < 0.05 was considered significant.

	logFC	logCPM	*p*‐value
hsa‐miR‐92a‐1‐5p	−2.59249230	4.51301526	0.0084022
hsa‐miR‐411‐3p	−2.57668920	5.46026155	0.0303435
hsa‐miR‐1254	−2.19662800	3.4468902	0.0323731
hsa‐miR‐889‐3p	−1.74577420	6.19610291	0.0260209
hsa‐miR‐323a‐3p	−1.36009400	7.20670985	0.0192232
hsa‐miR‐379‐5p	−1.22418870	9.49526416	0.0292624
hsa‐miR‐369‐3p	−1.07979050	8.32588798	0.0147740
hsa‐miR‐487b‐3p	−0.84249410	9.39542435	0.0489313
hsa‐miR‐335‐5p	−0.79584640	11.1074475	0.0401338
hsa‐miR‐92a‐3p	0.73659112	15.6989821	0.0476906
hsa‐miR‐320b	0.81805350	9.03565081	0.0235586
hsa‐miR‐744‐5p	0.90153749	10.898169	0.0245162
hsa‐miR‐3184‐5p	0.93371360	9.14392537	0.0418352
hsa‐miR‐185‐3p	0.93620145	8.51063252	0.0249045
hsa‐miR‐320a	0.97575443	14.4077648	0.0073678
hsa‐miR‐23a‐5p	1.07398104	8.12169886	0.0484872
hsa‐miR‐486‐3p	1.17431950	11.0462275	0.0479907
hsa‐miR‐193a‐5p	1.18912663	9.07039778	0.0243180
hsa‐miR‐3591‐3p	1.53886969	9.58540681	0.0420058
hsa‐miR‐1307‐5p	1.62539864	5.74102969	0.0239637
hsa‐miR‐122‐5p	1.74658218	9.90241548	0.0169855
hsa‐miR‐450b‐5p	1.78405584	6.37459753	0.0342440
hsa‐miR‐4732‐5p	1.84254735	6.17336638	0.0346117
hsa‐miR‐1290	2.69838719	7.57378949	0.0018963

### 3.3 miRNAs exclusively upregulated in Okinawan nonagenarians demonstrate predictive regulatory interactions in key nutrient sensing pathways impacted with age

**FIGURE 3:**
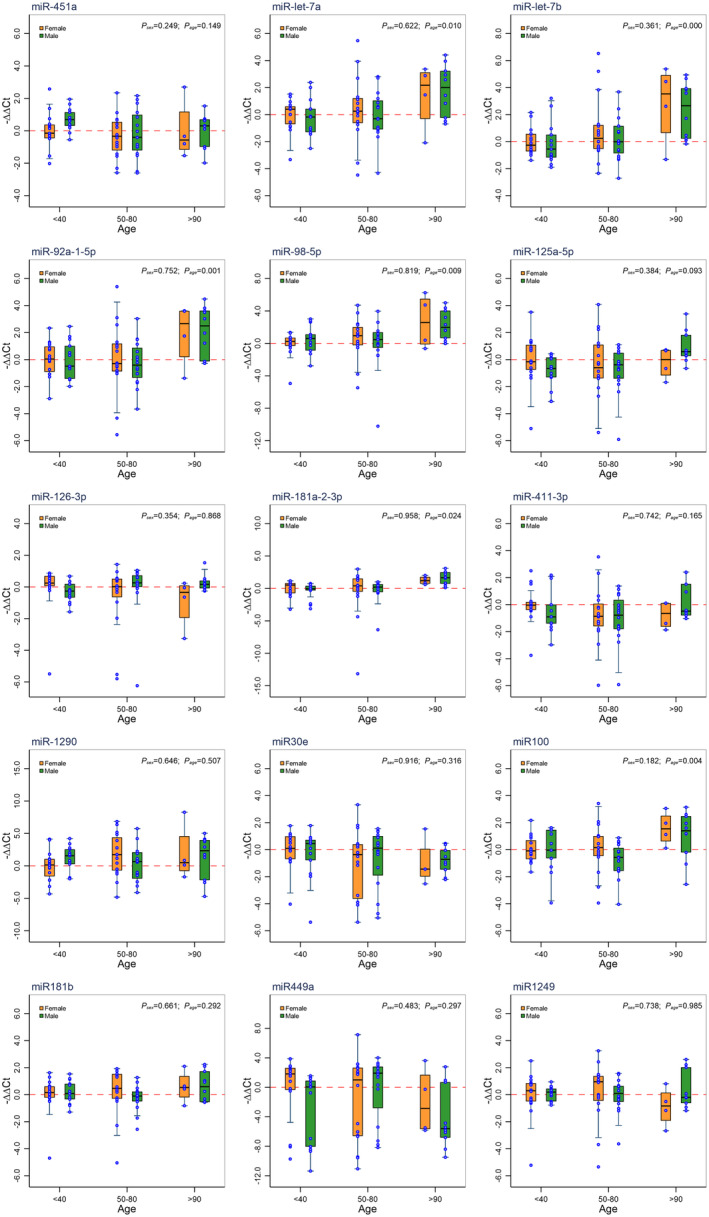
Boxplots demonstrating expression of 15 miRNAs quantified through RT‐qPCR and their association with sex and age. The expression of the 15 miRNAs were measured as −∆∆Ct normalized using the housekeeping miRNA miR‐16 and females of <40 years of age as a baseline subgroup. Multi‐factor ANOVA analysis was performed to test the partial effect of sex and age on the expression of each miRNA. The results suggest that the expressions of miR‐let‐7a, miR‐let‐7b, miR‐92a‐1‐5p, miR‐98‐5p, miR‐181a‐2‐3p, and miR100 are significantly up‐regulated in individuals of >90 years of age with the longevity genotype (TG alleles) (*p* = 0.010, *p* < 0.0001, *p* = 0.001, *p* = 0.009, *p* = 0.024 and *p* = 0.004, respectively), and that the expressions of the 15 studied miRNAs are not significantly associated with sex (refer to Figures S2–S5 for sex, age, and genotype associations evaluated collectively and individually).

**FIGURE 4:**
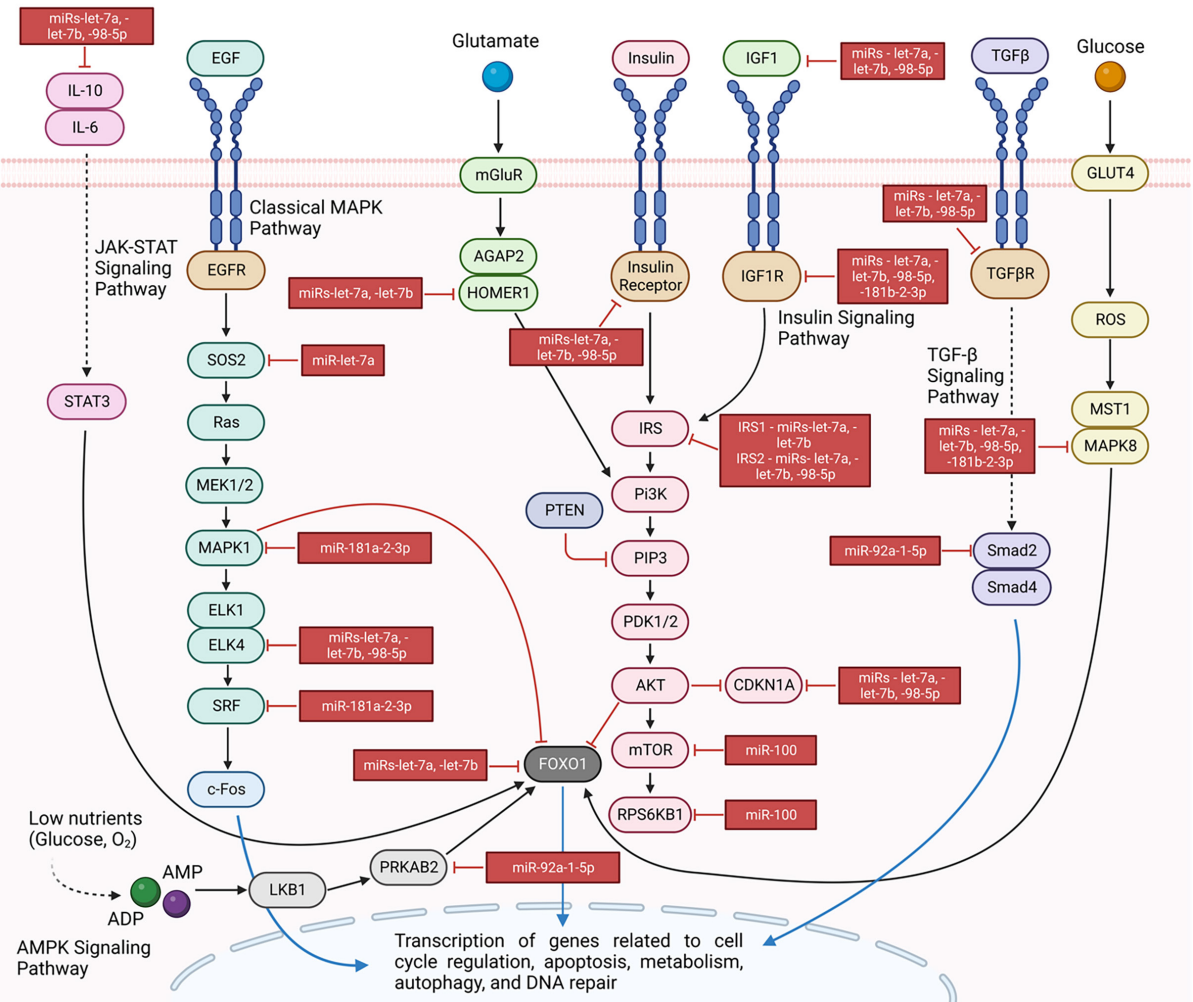
Schematic representation of predicted pathway interactions of microRNAs upregulated in individuals >90 years of age, present only in those subjects with the FOXO3 longevity genotype (GT). Inhibitory effects of microRNAs depicted represent post‐transcriptional regulation of gene expression. Figure was made with BioRender and KEGG pathways. Pathway interactions were derived from DIANA Tools and predicted gene targets were determined using the microT‐CDS target prediction algorithm (Vlachos et al., 2015). For full list of pathways, refer to Table 1. For miRNA‐gene interactions, refer to Figure S6.

Based on the results of sRNA‐seq and due to the narrowed range of samples per group post‐QC analysis, qPCR was performed using all samples (total *n* = 76) on 10 candidate miRNAs: miR‐451a, let‐7a, let‐7b, miR‐92a‐1‐5p, miR‐98‐5p, miR‐125a‐5p, miR‐126‐3p, miR‐181a‐2‐3p, miR‐411‐3p, and miR‐1290 demonstrating lower *p*‐values and higher fold change amounts in the comparisons made with RNASeq data. Moreover, we quantified the expression of 5 additional miRNAs: miR‐30e, miR‐100, miR‐181b‐5p, miR‐449a‐5p, and miR‐1249 of interest based on our previous studies evaluating pro‐longevity miRNAs in humans and mice with congenital growth hormone deficiencies (Noureddine et al., 2023; Saccon et al., 2021; Schneider et al., 2017). Relative expression normalized to the housekeeping miRNA miR‐16 suggests let‐7a, let‐7b, miR‐92a‐1‐5p, miR‐98‐5p, miR‐181a‐2‐3p, and miR‐100 are significantly and uniquely upregulated (Figure 3, *p* = 0.010, <0.0001, 0.001, 0.009, 0.024, and 0.004, respectively, respectively) in nonagenarians and that the miRNAs validated through RT‐qPCR do not demonstrate significant sex or genotype associations (Figure 3 and Figures S2–S5). Pathway analysis of the uniquely upregulated miRNAs in nonagenarians revealed predicted interactions in signaling pathways regulating pluripotency of stem cells as well as TGF‐β, FoxO, AMPK, Pi3k‐Akt, and MAPK signaling pathways (Table 2). More specifically, gene‐target analysis reveals let‐7a, let‐7b, miR‐92a‐1‐5p, miR‐98‐5p, miR‐181a‐2‐3p, and miR‐100 regulate a variety of key genes implicated with aging including *interleukin‐10* (*Il‐10*), *Sos2*, *Mapk1*, *mTOR*, *Cdkn1a*, *Irs*, *Igf‐1*, and *Foxo1* as summarized by Figure 4 and reiterated in Figure S6.

**TABLE 2:** Predicted pathway interactions of significantly up‐regulated microRNAs miR‐let‐7a, miR‐let‐7b, miR‐92a‐1‐5p, miR‐98‐5p, miR‐181a‐2‐3p, and miR100 in individuals of age >90. Pathway interactions with p‐value <0.05 were derived using DIANA Tools (Vlachos et al., 2015).

KEGG pathway	*p*‐value	#genes	#miRNAs
Signaling pathways regulating pluripotency of stem cells	7.72418656617E‐10	43	9
ECM‐receptor interaction	3.95899809297E‐07	18	7
Proteoglycans in cancer	2.32999005982E‐06	42	9
TGF‐beta signaling pathway	4.44598698368E‐05	23	8
Mucin type O‐Glycan biosynthesis	5.31064644059E‐05	7	6
Adherens junction	0.000381407252262	24	8
Wnt signaling pathway	0.000575629506475	31	9
Arrhythmogenic right ventricular cardiomyopathy (ARVC)	0.000594667713859	18	8
Pathways in cancer	0.00175326604254	71	9
FoxO signaling pathway	0.00806319554554	30	8
Chronic myeloid leukemia	0.00987802605257	15	8
Basal cell carcinoma	0.0167731126961	15	9
AMPK signaling pathway	0.0177380858414	27	9
Axon guidance	0.0186489599341	25	7
Glutamatergic synapse	0.0187558149041	21	7
Nicotine addiction	0.0187558149041	8	7
Amoebiasis	0.0187558149041	20	8
Transcriptional misregulation in cancer	0.0187558149041	34	8
PI3K‐Akt signaling pathway	0.0187558149041	62	9
Lysine degradation	0.0207431323703	8	7
Prostate cancer	0.0299131763703	21	8
Glioma	0.0498877979777	14	8
MAPK signaling pathway	0.0498877979777	44	9
Focal adhesion	0.0498877979777	38	9

## 4 DISCUSSION AND CONCLUSION

miRNAs are important regulators of biological and metabolic processes (Kinser & Pincus, 2019). Through their ability to bind to sequence‐compatible mRNAs and repress translation, these small noncoding RNAs can regulate a variety of signaling pathways and cellular mechanisms (Dimmeler & Nicotera, 2013). miRNA expression patterns are altered by several conditions and play a key role in organismal development. Aging and certain biological processes can affect these patterns resulting in modified pathway regulation, ultimately contributing to de‐regulated nutrient sensing, altered metabolism, insulin resistance, senescence, and increased inflammation with age (Noureddine et al., 2022; Victoria et al., 2015). Our sRNA‐sequencing analysis of circulating miRNAs in young (<40 years of age), middle‐aged and elderly (50–80 years of age), and nonagenarian (>90 years of age) individuals demonstrated vastly different miRNA expression profiles across age groups. Specifically, our findings suggest increased regulation of genes involved in FoxO, Ras, TGF‐β, Pi3K‐Akt, MAPK, mTOR, insulin signaling, and type II diabetes mellitus in the younger cohort when compared to the middle aged and elderly Okinawan individuals. Surprisingly, these pathways appear to be upregulated in the younger cohort when compared to individuals >90 years of age, suggesting that Okinawan nonagenarians exhibit improved regulation of pathways implicated with aging. Similarly, the middle‐aged and elderly cohort demonstrate downregulation of miRNAs predicted to be involved in regulating nutrient sensing pathways. Hence, our findings support the notion that miRNA expression patterns differ with age and that Okinawan nonagenarians exhibit improved health span by way of increased regulation of key nutrient sensing and metabolic pathways. Similarly, our genotypic analysis evaluating associations between SNPs in the *FOXO3* gene and miRNA expression patterns revealed individuals with from the TG genotype have improved regulation of age‐associated and metabolism‐related pathways. Furthermore, RT‐qPCR analysis on 10 miRNAs displaying unique expression patterns in nonagenarians compared to middle‐elderly aged and younger individuals as well as 5 miRNAs of interest to the field of aging research revealed 5 uniquely upregulated miRNAs in circulation of individuals >90 years of age.

Prior studies have demonstrated varying roles of let‐7 in aging, with a perceived increase in insulin resistance, as well as onset of cardiovascular and age‐related diseases associated with overexpression of let‐7 (Wang et al., 2022). However, loss of let‐7 has conversely been shown to reduce lifespan in *C. elegans* (Xu et al., 2014). Hence, the role of this miRNA family in aging appears to be multifaceted. Our findings demonstrate an increase in the let‐7a and let‐7b miRNAs encoded by the let‐7 gene. Based on our predictive pathway analysis, these miRNAs target various components of the insulin/IGF‐1 and mammalian target of rapamycin (mTOR) pathways. These pathways are notoriously de‐regulated with age and have been widely implicated in promoting aging and onset of age‐related diseases (López‐Otín et al., 2013). Inhibition of insulin/IGF‐1 and mTOR signaling pathways can extend lifespan with prior research demonstrating improved lifespan directly attributed to inhibition of mTOR (Johnson et al., 2013). Some studies have also established that let‐7a‐targeted regulation of important genes such as *P66shc* leads to delayed senescence in human dermal fibroblasts (Xu et al., 2014). Coupled with the apparent role in targeting nutrient sensing genes *Igf‐1*, *Igf‐1‐receptor* (*Igf1r*), *insulin receptor substrate* (*Irs*) *1 and 2, Homer scaffold protein 1* (*Homer1*) as well as senescence‐related genes *Cdkn1a* (*p21* ^*cip1*^) and *Tgf‐*β *receptor* (*Tgf*β*r*), microRNAs let‐7a and let‐7b exhibit important regulatory contributions to aging and are uniquely upregulated in Okinawan nonagenarians, warranting further investigations into their roles in metabolism and longevity.

Similarly, miR‐98‐5p—also encoded by the let‐7 gene—predictively targets *Il‐10, insulin receptor*, *Igf‐1*, *Igf‐1R*, *Tgf‐*β*R*, *Mapk8*, *Irs2*, *Cdkn1a*, and *Elk4*. Importantly, this miRNA appears to be involved in the same nutrient sensing, senescence, and pro‐inflammatory pathways as let‐7a and let‐7b (Figure 4). Prior studies have demonstrated that downregulation of miR‐98‐5p is associated with onset of type II diabetes, primarily mediated by the upregulation in gene‐target *Ppp1R15*β (Khan et al., 2020). Furthermore, miR‐98‐5p has been confirmed to target *Igf‐2 binding protein* (*Igf2bp1*), which leads to inhibition of the Pi3K‐AKT pathway, a nutrient sensing pathway typically associated with promoting aging through the activation of mTOR (Noureddine et al., 2023; Stallone et al., 2019; Wang et al., 2020). Hence, miRs‐98‐5p, −let‐7a, and −let‐7b's apparent roles in metabolism may contribute to the observed enhanced insulin sensitivity and overall improved health of the nonagenarian cohort.

In addition to metabolism, lifespan and health span are both impacted by the accrual of senescent cells with age. As a hallmark of aging, cellular senescence contributes to accelerated aging and chronic inflammation primarily through the senescence‐associated secretory phenotype (SASP) (López‐Otín et al., 2013). This stress‐response can be triggered by TGF‐β signaling which involves the activation of the Smad transcription complex (comprised of Smad2 and Smad4) necessary for mediating the signaling cascade that promotes the transcription of genes related to cellular proliferation, senescence, and the production of the SASP (Tominaga & Suzuki, 2019). TGF‐β also promotes radical oxygen species (ROS) production, DNA damage, as well as the suppression of telomerase activity both dependently and independently of Smad activation (Liu & Desai, 2015; Tominaga & Suzuki, 2019). Taken together, TGF‐β signaling can contribute significantly to aging and age‐associated diseases. Interestingly, miR‐92a‐1‐5p, significantly upregulated in circulation in Okinawan nonagenarians, is predicted to target *Smad2* post‐transcriptionally, thereby eliciting its important potential function in regulating TGF‐β signaling and cellular senescence. However, miR‐92a‐1‐5p has also been predicted to target *Prka*β*2*, the gene encoding the human AMPK‐β2 subunit—a nutrient‐sensing gene that plays an important role in AMPK signaling (Ziegler et al., 2020). AMPK activation/responsiveness typically declines with age, leading to reduced autophagy and increased oxidative stress (Stancu, 2015). Hence, the role of miR‐92a‐1‐5p in aging requires further investigation specifically into its involvement in senescence as well as its potential regulatory function in AMPK signaling.

Similarly, miR‐181a‐2‐3p is expected to be involved in regulating senescent cell survival through its ability to bind and inhibit the activity of serum response factor (*srf*). SRF binds the ETS (Erythroblast Transformation Specific) domain of transcription factor serum response element (SRE) to mediate gene expression of essential MAPK signaling genes (Whitmarsh et al., 1995). MAPKs promote key factors necessary for the persistence of senescent cells, such as growth arrest, cell survival, and the SASP, acting as important mediators of senescent cell survival (Anerillas et al., 2020). Taken together, upregulation of miR‐92a‐1‐5p and miR‐181a‐2‐3p could be contributing to the enhanced longevity of the nonagenarian cohort through regulation of cellular senescence and pathways involved in maintaining both the SASP and SnC survival.

mTOR has also been widely implicated in accelerating aging and aggravating age‐related pathologies primarily through its ability to promote increased cell survival and proliferation coupled with inhibition of autophagy. Increased mTOR expression (through the activation of the Pi3K‐AKT signaling cascade) can lead to protein aggregates and mitochondrial dysfunction with subsequent ROS production and cellular stress (Stallone et al., 2019). Hence, mTOR is a significant pathway to regulate and an important target for age‐related interventions. Further, it has been well established that targeting mTOR successfully decelerates aging and reduces senescent cell accumulation (Weichhart, 2019). miR‐100 targets and suppresses the translation of *mTOR* post‐transcriptionally, therefore its therapeutical potential has been explored (Saccon et al., 2021). Prior research has also demonstrated that miR‐100 overexpression in mice attenuates high‐fat diet‐induced weight gain, liver steatosis, and metabolic syndrome (Smolka et al., 2021). These mice also have improved insulin sensitivity and energy expenditure, possibly mediated by miR‐100. Of interest to this study, miR‐100 was found to be uniquely upregulated in circulation of the Okinawan nonagenarian cohort, suggesting that these individuals exhibit improved regulation of mTOR and nutrient sensing pathways, a notion that may justify their overall improved metabolism and insulin sensitivity.

In short, our data demonstrates potential links between miRNAs let‐7a, let‐7b, miR‐92a‐1‐5p, miR‐98‐5p, miR‐181a‐2‐3p, and miR‐100 and key age‐related pathways that are implicated in affecting longevity. Our study also validates the current literature regarding the variability in expression of miRNAs with age and genotype, suggesting that specific age‐impacted miRNAs could function as biomarkers to predict changes in regulation of age‐related pathways and metabolism. Furthermore, there is growing interest in the potential for miRNAs to serve as therapeutics in the treatment of age‐related diseases due to the capacity of these small RNAs in regulating a variety of signaling pathways. Hence, identifying candidate miRNAs for further study, as presented in this study, is crucial for the advancement of novel drug developments and diagnostic methods. Moreover, the findings demonstrated in this study provide added support toward the enhanced metabolism, insulin sensitivity, health span and lifespan observed in Okinawan nonagenarians based on the predicted regulatory functions of upregulated miRNAs further emphasizing the importance of metabolism and nutrient sensing pathways in aging. Our findings also support the correlation between *FOXO3* genetic carriers of the G allele and improved metabolism, as observed by the genotype and miRNA associations predictively demonstrating improved regulation of nutrient sensing pathways in individuals with TG alleles versus TT alleles. Our study limitations include variable age distribution across males and females as well as sample size per group. Future considerations include narrowing age ranges per cohort to further specify changes in miRNA expression with age as well as verifying the miRNA pathway interactions presented in this study to solidify their potential pro‐longevity functions. It would also be interesting to explore their capacity to act as biomarkers to predict regulation patterns of key aging pathways. Overall, our results provide a strong rationale for added investigation into the mechanistic functions of the miRNAs uniquely upregulated in circulation in Okinawan nonagenarians and their potential to be used in future therapies.

## AUTHOR CONTRIBUTIONS

*Concept and design, acquisition of data, analysis, interpretation of data, manuscript original draft, manuscript revision*: Sarah Noureddine. *Interpretation of data, analysis, manuscript original draft, manuscript revision*: Augusto Schneider. *Acquisition of data, analysis, manuscript revision*: Sydney Strader. *Data analysis, interpretation of data, manuscript original draft, manuscript revision*: Xiang Zhu. *Funding acquisition, analysis, manuscript revision*: Joseph Dhahbi. *Concept and design, acquisition of samples, cohort recruitment, manuscript revision*: Richard Allsopp. *Concept and design, acquisition of samples, cohort recruitment, manuscript revision*: D. Craig Willcox. *Interpretation of data, manuscript revision*: Timothy A Donlon. *Concept and design, acquisition of samples, cohort recruitment, manuscript revision*: Michio Shimabukuro. *Acquisition of samples, cohort recruitment, manuscript revision*: Moritake Higa. *Concept and design, acquisition of samples, cohort recruitment, manuscript revision*: Makoto Suzuki. *Concept and design, acquisition of samples, manuscript revision*: Trevor Torigoe. *Interpretation of data, manuscript revision*: Sarah Ashiqueali. *Concept and design, acquisition of samples, cohort recruitment, manuscript revision*: Hariom Yadav. Concept and design, acquisition of samples, cohort recruitment, manuscript revision: Bradley J. Willcox. *Concept and design, funding acquisition, acquisition of data, analysis, interpretation of data, manuscript original draft, manuscript revision*: Michal M. Masternak.

## CONFLICT OF INTEREST STATEMENT

The authors declare no conflicts of interest.

## Supporting information


Data S1:

## Data Availability

The data that support the findings of this study are available in the supplementary material of this article. Supporting data has also been registered with the BioProject database (BioProject ID: PRJNA1054150).
