# Supplementary material for: Circulating microRNA profile of long‐lived Okinawans identifies novel potential targets for optimizing lifespan and health span
Source: Aging Cell. 2024 May 15;23(8):e14191. doi: 10.1111/acel.14191 (PMC11320357; doi:10.1111/acel.14191)
Supplement: Supplementary file 1 — Data S1: [file ACEL-23-e14191-s001.docx]

**Supporting Data**

**Circulating microRNA profile of long-lived Okinawans identifies novel potential targets for optimizing lifespan and health span.**

Sarah Noureddine^1^, Augusto Schneider^2^, Sydney Strader^1^, Xiang Zhu^1^, Joseph Dhahbi^3^, Richard Allsopp^4,5,6^, D. Craig Willcox^5,6,7^, Timothy A. Donlon^5,8^, Michio Shimabukuro^6,9^, Moritake Higa^10^, Makoto Suzuki^6^, Trevor Torigoe^4^, Sarah Ashiqueali^1^, Hariom Yadav^11^, Bradley J. Willcox^5,6,12*^, and Michal M. Masternak^1,13*^

^1^University of Central Florida College of Medicine, Burnett School of Biomedical Sciences, Orlando, FL 32827, USA.

^2^Faculdade de Nutrição, Universidade Federal de Pelotas, Pelotas, Brazil.

^3^Department of Medical Education, School of Medicine, California University of Science & Medicine, Colton, CA, USA.

^4^Institute for Biogenesis Research, John A. Burns School of Medicine, University of Hawai’i, HI, USA.

^5^Center of Biomedical Research Excellence for Translational Research on Aging, Kuakini Medical Center, Honolulu, HI, USA.

^6^Okinawa Research Center for Longevity Science, Urasoe, Okinawa, Japan

^7^Department of Human Welfare, Okinawa International University, Ginowan, Okinawa, Japan

^8^Department of Cell and Molecular Biology, John A. Burns School of Medicine, University of Hawai’i, HI, USA.
^9^Department of Diabetes, Endocrinology and Metabolism, Fukushima Medical University, School of Medicine, Fukushima, Japan.
^10^Diabetes and Life-Style Related Disease Center, Tomishiro Central Hospital, Tomishiro, Okinawa, Japan.

^11^USF Center for Microbiome Research, Microbiomes Institute, University of South Florida Morsani College of Medicine, Tampa, Florida, USA.

^12^Department of Geriatric Medicine, John A. Burns School of Medicine, University of Hawai’i, HI, USA.
^13^Department of Head and Neck Surgery, Poznan University of Medical Sciences, Poznan, Poland

**Corresponding authors**

Michal M. Masternak
University of Central Florida, Burnett School of Biomedical Sciences, College of Medicine
6900 Lake Nona Blvd, Orlando, FL 32827, USA.
E-mail: [michal.masternak@ucf.edu](mailto:michal.masternak@ucf.edu)

Bradley J. Willcox

Department of Geriatric Medicine, John A. School of Medicine, University of Hawaii

Kuakini Medical Center Campus, HPM-9

347 N. Kuakini Street, Honolulu, HI 96817, USA

E-mail: [willcox@hawaii.edu](mailto:willcox@hawaii.edu)

**Supplemental Table 1.** Characteristics of included participants.

| **Supplemental Table 1. General Characteristics of Participants** | | |
| --- | --- | --- |
|  | Missing | Total (n=76) |
| Men, n (%) | 0 | 36 (47.3) |
| Age, years (y) | 0 | 58.6 ± 25.3 |
| Age, years [range] | 0 | [19-102] |
| **Biological / Social** | | |
| Body weight, kg | 3 | 62.2 ± 15.2 |
| Body weight, kg [range] | 3 | [31.4 – 115.0] |
| Body mass index, kg/m^2^ | 3 | 24.8 ± 4.4 |
| Body mass index, kg/m^2^ [range] | 3 | [16.0 – 37.1] |
| Systolic blood pressure, mmHg | 0 | 131 ± 15 |
| Diastolic blood pressure, mmHg | 0 | 74 ± 10 |
| Current smoker, n (%) | 10 | 9 (13.6) |
| Current drinker, n (%) | 10 | 19 (28.8) |
| **Prevalent Disease** | | |
| Diabetes mellitus, n (%) | 2 | 37 (50.0) |
| Hypertension, n (%) | 2 | 41 (55.4) |
| Ischemic heart disease, n (%) | 2 | 11 (14.9) |
| Cerebrovascular disease, n (%) | 2 | 13 (17.6) |
| Cancer, n (%) | 2 | 13 (17.6) |
| Data are presented as mean ± SD, n (%), or range. | | |

**
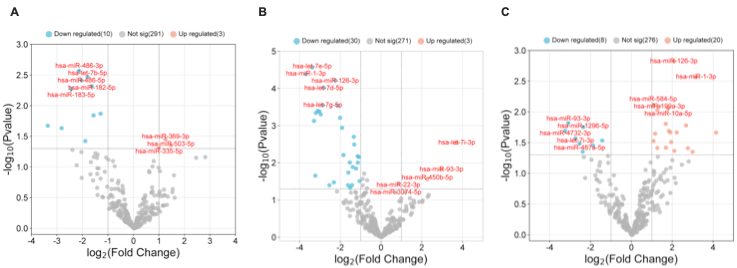
**

**Supplemental Figure 1.** Volcano plots. **A,** depicts miRNA expression differences between young (<40 years of age) individuals and nonagenarians (=≥ 90 years of age). **B,** depicts differences between middle-aged (50-80 years of age ) individuals and nonagenarians (=≥ 90 years of age). **C,** depicts differences between young (<40 years of age) and middle-aged (50-80 years of age) individuals. log_2_ Fold Change indicates the mean expression level for each miRNA. Each dot represents one miRNA.

**Supplemental Table 2.** List of TaqMan MicroRNA Assays used for quantifying the expression of miRNAs of interest through qRT-PCR.

| **miRNA** | **TaqMan MicroRNA Assay** |
| --- | --- |
| **miR-16-5p** | 477860_mir hsa-miR-16-5p |
| **miR-451a** | 478107_mir hsa-miR451a |
| **miR-let-7a** | 478575_mir hsa-let-7a-5p |
| **miR-let-7b** | 478575_mir hsa-let-7b-5p |
| **miR-92a-1-5p** | 479205_mir hsa-miR-92a-1-5p |
| **miR-98-5p** | 478590_mir hsa-miR-98-5p |
| **miR-125a-5p** | 477884_mir hsa-miR-125a-5p |
| **miR-126-3p** | 477887_mir hsa-126-3p |
| **miR-181a-2-3p** | 479478_mir hsa-miR-181a-2-3p |
| **miR-411-3p** | 479536_mir hsa-411-3p |
| **mIR-1290** | 477895_mir hsa-miR-1290 |
| **miR-30e** | 479235_mir hsa-miR-30e-5p |
| **miR-100-5p** | 478224_mir hsa-miR-100-5p |
| **miR-181b-5p** | 478583_mir hsa-miR-181b-5p |
| **miR-449a-5p** | 478561_mir hsa-miR-449a-5p |
| **miR-1249** | 479554_mir hsa-miR-1249 |

**Supplemental Table 3.** Differentially expressed miRNAs in young individuals (<40 years of age) compared to nonagenarians ( =≥ 90 years of age). logFC indicates log fold change and logCPM represents log counts per million indicative of expression levels; p-value < 0.05 was considered significant.

|  | **logFC** | **logCPM** | **p-value** |
| --- | --- | --- | --- |
| **hsa-miR-1246** | -3.357817 | 5.688994 | 0.021094 |
| **hsa-miR-3679-5p** | -2.814474 | 4.101812 | 0.023149 |
| **hsa-miR-183-5p** | -2.444512 | 8.005034 | 0.005322 |
| **hsa-miR-486-3p** | -2.132970 | 11.046220 | 0.002715 |
| **hsa-miR-486-5p** | -2.041899 | 15.775254 | 0.003856 |
| **hsa-let-7a-5p** | -1.888451 | 15.835109 | 0.037815 |
| **hsa-let-7b-5p** | -1.801198 | 13.785783 | 0.003374 |
| **hsa-miR-182-5p** | -1.632433 | 10.581014 | 0.004804 |
| **hsa-miR-451a** | -1.564041 | 16.195127 | 0.014375 |
| **hsa-miR-342-3p** | -1.288671 | 10.561392 | 0.013458 |
| **hsa-miR-335-5p** | 1.002761 | 11.107447 | 0.048344 |
| **hsa-miR-369-3p** | 1.256677 | 8.325891 | 0.035782 |
| **hsa-miR-503-5p** | 1.467429 | 6.979356 | 0.044526 |

**Supplemental Table 4.** Predicted pathway interactions of significantly down-regulated microRNAs in young individuals (<40 years of age) compared to nonagenarians. Pathway interactions with p-value < 0.05 were derived using DIANA Tools (Vlachos et al., 2015).

| **KEGG pathway** | **p-value** | **#genes** | **#miRNAs** |
| --- | --- | --- | --- |
| **ECM-receptor interaction** | 1.16157399523E-09 | 24 | 9 |
| **Proteoglycans in cancer** | 4.84587427102E-06 | 62 | 10 |
| **TGF-beta signaling pathway** | 3.28273261376E-05 | 30 | 9 |
| **ErbB signaling pathway** | 0.000249682470768 | 32 | 10 |
| **PI3K-Akt signaling pathway** | 0.000249682470768 | 98 | 10 |
| **GABAergic synapse** | 0.000372842135028 | 23 | 9 |
| **Melanoma** | 0.00175125532715 | 26 | 9 |
| **Prostate cancer** | 0.00178244084627 | 32 | 9 |
| **Signaling pathways regulating pluripotency of stem cells** | 0.00206210600179 | 42 | 8 |
| **AMPK signaling pathway** | 0.00206210600179 | 42 | 9 |
| **Glioma** | 0.00206210600179 | 22 | 9 |
| **Pathways in cancer** | 0.0039640919932 | 101 | 10 |
| **Vasopressin-regulated water reabsorption** | 0.00474879835475 | 16 | 6 |
| **Regulation of actin cytoskeleton** | 0.00474879835475 | 61 | 9 |
| **Bacterial invasion of epithelial cells** | 0.00474879835475 | 25 | 10 |
| **Glutamatergic synapse** | 0.00530065549612 | 32 | 9 |
| **Thyroid hormone signaling pathway** | 0.00587865833694 | 31 | 9 |
| **Nicotine addiction** | 0.00658530471271 | 14 | 9 |
| **FoxO signaling pathway** | 0.00658530471271 | 39 | 10 |
| **Estrogen signaling pathway** | 0.00658530471271 | 26 | 10 |
| **Morphine addiction** | 0.00785519449442 | 24 | 8 |
| **Phosphatidylinositol signaling system** | 0.00840951428377 | 22 | 8 |
| **Axon guidance** | 0.00840951428377 | 36 | 8 |
| **Colorectal cancer** | 0.00840951428377 | 22 | 8 |
| **Chronic myeloid leukemia** | 0.00840951428377 | 23 | 9 |
| **Focal adhesion** | 0.00840951428377 | 59 | 10 |
| **Adrenergic signaling in cardiomyocytes** | 0.0105784286868 | 39 | 10 |
| **Serotonergic synapse** | 0.0118538611803 | 30 | 9 |
| **Neurotrophin signaling pathway** | 0.0140769279262 | 38 | 9 |
| **Hippo signaling pathway** | 0.0159389344953 | 44 | 9 |
| **Transcriptional misregulation in cancer** | 0.0159389344953 | 46 | 9 |
| **Amoebiasis** | 0.0323745452539 | 29 | 9 |
| **Wnt signaling pathway** | 0.0346853449631 | 39 | 9 |
| **mTOR signaling pathway** | 0.0360833527328 | 21 | 9 |
| **Viral carcinogenesis** | 0.036811284437 | 38 | 10 |
| **Retrograde endocannabinoid signaling** | 0.041699446169 | 31 | 9 |
| **MAPK signaling pathway** | 0.0453425027847 | 65 | 10 |
| **Endometrial cancer** | 0.0470583066406 | 18 | 8 |

**Supplemental Table 5.** Predicted pathway interactions of significantly up-regulated microRNAs in young individuals (<40 years of age) compared to nonagenarians. Pathway interactions with p-value < 0.05 were derived using DIANA Tools (Vlachos et al., 2015).

| **KEGG pathway** | **p-value** | **#genes** | **#miRNAs** |
| --- | --- | --- | --- |
| **Signaling pathways regulating pluripotency of stem cells** | 3.80609377811E-08 | 32 | 2 |
| **Glycosaminoglycan biosynthesis - chondroitin sulfate / dermatan sulfate** | 0.000277571023628 | 5 | 1 |
| **Drug metabolism - cytochrome P450** | 0.000277571023628 | 6 | 1 |
| **TGF-beta signaling pathway** | 0.000277571023628 | 22 | 2 |
| **Proteoglycans in cancer** | 0.000277571023628 | 33 | 2 |
| **Melanoma** | 0.000277571023628 | 18 | 2 |
| **Pathways in cancer** | 0.000476707085855 | 55 | 3 |
| **Renal cell carcinoma** | 0.000476707085855 | 18 | 3 |
| **Glioma** | 0.000614982971746 | 14 | 3 |
| **Prolactin signaling pathway** | 0.00149490316792 | 15 | 3 |
| **Morphine addiction** | 0.00152174030121 | 19 | 1 |
| **GABAergic synapse** | 0.00171515488204 | 11 | 1 |
| **Non-small cell lung cancer** | 0.0043705310295 | 12 | 2 |
| **Rap1 signaling pathway** | 0.0043705310295 | 36 | 3 |
| **Prostate cancer** | 0.00568088416137 | 18 | 2 |
| **Dorso-ventral axis formation** | 0.0093510829057 | 9 | 2 |
| **Acute myeloid leukemia** | 0.00981906974134 | 12 | 2 |
| **Thyroid cancer** | 0.0105500698676 | 6 | 1 |
| **Maturity onset diabetes of the young** | 0.0114478425329 | 5 | 1 |
| **Axon guidance** | 0.0114478425329 | 25 | 2 |
| **Chronic myeloid leukemia** | 0.0165897264297 | 16 | 2 |
| **Progesterone-mediated oocyte maturation** | 0.0199134370213 | 18 | 2 |
| **Endometrial cancer** | 0.0199134370213 | 12 | 2 |
| **Hippo signaling pathway** | 0.0199134370213 | 31 | 3 |
| **Ubiquitin mediated proteolysis** | 0.0249135777212 | 24 | 3 |
| **Ras signaling pathway** | 0.0295700371103 | 33 | 3 |
| **FoxO signaling pathway** | 0.0331407801986 | 25 | 2 |
| **Long-term potentiation** | 0.0331407801986 | 14 | 2 |
| **Estrogen signaling pathway** | 0.0344985952956 | 14 | 3 |

**Supplemental Table 6.** Differentially expressed miRNAs in middle-aged individuals (50-80 years of age) compared to nonagenarians (=≥ 90 years of age). logFC indicates log fold change and logCPM represents log counts per million indicative of expression levels; p-value < 0.05 was considered significant.

|  | **logFC** | **logCPM** | **p-value** |
| --- | --- | --- | --- |
| **hsa-miR-1-3p** | -3.6902258 | 9.127073 | 4.139E-05 |
| **hsa-let-7e-5p** | -3.3817408 | 8.884078 | 2.727E-05 |
| **hsa-miR-574-5p** | -3.2963115 | 7.402284 | 7.419E-04 |
| **hsa-miR-411-5p** | -3.2318054 | 7.178754 | 2.222E-02 |
| **hsa-let-7a-5p** | -3.2044483 | 15.835109 | 4.525E-04 |
| **hsa-miR-98-5p** | -3.1153503 | 11.561380 | 3.993E-04 |
| **hsa-let-7f-5p** | -3.0269706 | 14.882005 | 4.168E-04 |
| **hsa-let-7c-5p** | -2.9646279 | 9.079548 | 4.963E-04 |
| **hsa-let-7g-5p** | -2.8600612 | 14.334736 | 2.817E-04 |
| **hsa-let-7d-5p** | -2.8148528 | 12.973841 | 9.511E-05 |
| **hsa-miR-369-5p** | -2.5358964 | 6.526131 | 4.015E-02 |
| **hsa-miR-340-3p** | -2.3156111 | 6.969712 | 3.360E-02 |
| **hsa-miR-126-3p** | -2.2538547 | 14.331914 | 6.030E-05 |
| **hsa-let-7i-5p** | -2.1403355 | 13.777577 | 2.889E-04 |
| **hsa-let-7b-5p** | -2.0120399 | 13.785783 | 6.137E-04 |
| **hsa-miR-10a-5p** | -1.9155909 | 9.225399 | 1.136E-03 |
| **hsa-miR-126-5p** | -1.8490771 | 9.319400 | 6.118E-03 |
| **hsa-miR-3613-5p** | -1.6239852 | 7.488751 | 4.005E-02 |
| **hsa-miR-26b-5p** | -1.6072112 | 9.506650 | 1.836E-02 |
| **hsa-miR-155-5p** | -1.5346637 | 8.955608 | 9.834E-03 |
| **hsa-miR-548j-5p** | -1.5188044 | 6.190069 | 4.689E-02 |
| **hsa-miR-200c-3p** | -1.4257467 | 7.143538 | 3.988E-02 |
| **hsa-miR-432-5p** | -1.3900175 | 9.802675 | 1.306E-02 |
| **hsa-miR-125a-5p** | -1.3387639 | 10.754532 | 1.983E-03 |
| **hsa-miR-199a-3p** | -1.3175715 | 11.471147 | 3.187E-03 |
| **hsa-miR-30b-5p** | -1.2273041 | 10.984221 | 1.429E-02 |
| **hsa-miR-26a-5p** | -1.1496378 | 15.603426 | 6.586E-03 |
| **hsa-miR-199b-3p** | -1.1399543 | 11.467428 | 9.951E-03 |
| **hsa-miR-181a-2-3p** | -1.0803986 | 8.609383 | 6.973E-03 |
| **hsa-miR-185-3p** | -1.0529748 | 8.510632 | 3.096E-02 |
| **hsa-miR-15b-5p** | -0.9051288 | 12.157983 | 2.708E-02 |
| **hsa-miR-30c-5p** | -0.9041016 | 11.068383 | 4.047E-02 |
| **hsa-miR-106b-3p** | -0.8279327 | 10.578517 | 1.956E-02 |
| **hsa-miR-584-5p** | -0.6871827 | 11.608487 | 4.844E-02 |
| **hsa-miR-30d-5p** | -0.6844717 | 12.824200 | 2.947E-02 |
| **hsa-miR-22-3p** | 0.8485136 | 14.705562 | 3.670E-02 |
| **hsa-miR-450b-5p** | 2.2658167 | 6.374554 | 2.754E-02 |
| **hsa-miR-93-3p** | 2.9607639 | 4.606638 | 1.520E-02 |
| **hsa-let-7i-3p** | 3.7115789 | 4.700847 | 2.862E-03 |

**Supplemental Table 7.** Predicted pathway interactions of significantly down-regulated microRNAs in middle-aged individuals (50-80 years of age) compared to nonagenarians. Pathway interactions with p-value < 0.05 were derived using DIANA Tools (Vlachos et al., 2015).

| **KEGG pathway** | **p-value** | **#genes** | **#miRNAs** |
| --- | --- | --- | --- |
| **Proteoglycans in cancer** | 2.16036685214E-12 | 116 | 31 |
| **Fatty acid biosynthesis** | 1.3072090655E-09 | 7 | 15 |
| **Hippo signaling pathway** | 7.56611121612E-08 | 83 | 32 |
| **Signaling pathways regulating pluripotency of stem cells** | 1.77211598226E-07 | 84 | 33 |
| **FoxO signaling pathway** | 1.45817478659E-06 | 84 | 32 |
| **Axon guidance** | 6.9366161184E-06 | 75 | 32 |
| **Glioma** | 8.46731431346E-06 | 40 | 29 |
| **Neurotrophin signaling pathway** | 8.46731431346E-06 | 75 | 33 |
| **Mucin type O-Glycan biosynthesis** | 2.30605561848E-05 | 16 | 26 |
| **Glycosaminoglycan biosynthesis - heparan sulfate / heparin** | 2.51579401338E-05 | 16 | 19 |
| **Ras signaling pathway** | 4.91463275617E-05 | 120 | 32 |
| **Prion diseases** | 9.67748191291E-05 | 11 | 11 |
| **Adrenergic signaling in cardiomyocytes** | 0.000144244856914 | 78 | 31 |
| **PI3K-Akt signaling pathway** | 0.000211731568978 | 173 | 32 |
| **Thyroid hormone signaling pathway** | 0.000214523550487 | 65 | 28 |
| **Oxytocin signaling pathway** | 0.000316063358464 | 87 | 32 |
| **Wnt signaling pathway** | 0.000422282330408 | 77 | 31 |
| **Glutamatergic synapse** | 0.000516711861233 | 62 | 31 |
| **MAPK signaling pathway** | 0.000520286437846 | 135 | 31 |
| **Pathways in cancer** | 0.000618957715338 | 197 | 32 |
| **mTOR signaling pathway** | 0.00066080775317 | 39 | 30 |
| **TGF-beta signaling pathway** | 0.000905606339201 | 46 | 32 |
| **Focal adhesion** | 0.000905606339201 | 109 | 32 |
| **Circadian entrainment** | 0.000984061441147 | 55 | 31 |
| **AMPK signaling pathway** | 0.000984061441147 | 70 | 32 |
| **ErbB signaling pathway** | 0.00172908029697 | 53 | 30 |
| **Long-term depression** | 0.00172908029697 | 35 | 30 |
| **Transcriptional misregulation in cancer** | 0.00202207564166 | 92 | 31 |
| **Chronic myeloid leukemia** | 0.00203037501454 | 43 | 29 |
| **Melanoma** | 0.00203037501454 | 42 | 30 |
| **Prostate cancer** | 0.00251363132534 | 51 | 30 |
| **Glycosaminoglycan biosynthesis - chondroitin sulfate / dermatan sulfate** | 0.00316438777114 | 11 | 16 |
| **Rap1 signaling pathway** | 0.00355932646154 | 107 | 32 |
| **Sphingolipid signaling pathway** | 0.00407530605667 | 64 | 29 |
| **Colorectal cancer** | 0.00420763789096 | 36 | 29 |
| **Long-term potentiation** | 0.00431980802145 | 40 | 31 |
| **Biotin metabolism** | 0.00461321277668 | 2 | 2 |
| **Morphine addiction** | 0.00580618519937 | 45 | 31 |
| **Renal cell carcinoma** | 0.00652407592013 | 40 | 30 |
| **Hypertrophic cardiomyopathy (HCM)** | 0.00652407592013 | 48 | 31 |
| **Viral carcinogenesis** | 0.00768541542332 | 80 | 30 |
| **N-Glycan biosynthesis** | 0.00999287912293 | 23 | 28 |
| **Arrhythmogenic right ventricular cardiomyopathy (ARVC)** | 0.010705515956 | 44 | 31 |
| **p53 signaling pathway** | 0.0108140981452 | 40 | 27 |
| **T cell receptor signaling pathway** | 0.0142421262342 | 56 | 29 |
| **Prolactin signaling pathway** | 0.0160680457159 | 40 | 28 |
| **Retrograde endocannabinoid signaling** | 0.0165641416059 | 52 | 31 |
| **Acute myeloid leukemia** | 0.0175320267662 | 33 | 29 |
| **Phosphatidylinositol signaling system** | 0.0183185240124 | 43 | 26 |
| **Basal cell carcinoma** | 0.018540316121 | 32 | 28 |
| **Dorso-ventral axis formation** | 0.0196857760484 | 18 | 27 |
| **Small cell lung cancer** | 0.0197649146122 | 46 | 27 |
| **Circadian rhythm** | 0.0198376889136 | 20 | 29 |
| **Pancreatic cancer** | 0.0198376889136 | 36 | 30 |
| **Cholinergic synapse** | 0.0198376889136 | 57 | 31 |
| **Dopaminergic synapse** | 0.0198376889136 | 71 | 31 |
| **Choline metabolism in cancer** | 0.0198376889136 | 55 | 31 |
| **Endometrial cancer** | 0.0241108705164 | 28 | 28 |
| **Gap junction** | 0.0295449741076 | 43 | 30 |
| **Melanogenesis** | 0.030256736613 | 51 | 31 |
| **Hedgehog signaling pathway** | 0.0355681733647 | 29 | 27 |
| **ECM-receptor interaction** | 0.0455049859165 | 39 | 30 |
| **cGMP-PKG signaling pathway** | 0.0472213398949 | 79 | 32 |
| **HIF-1 signaling pathway** | 0.0476693960556 | 54 | 31 |
| **Ubiquitin mediated proteolysis** | 0.0486821605865 | 70 | 32 |

| **KEGG pathway** | **p-value** | **#genes** | **#miRNAs** |
| --- | --- | --- | --- |
| **Glioma** | 0.000101676337692 | 17 | 3 |
| **Morphine addiction** | 0.000502235029711 | 16 | 3 |
| **Signaling pathways regulating pluripotency of stem cells** | 0.000502235029711 | 30 | 4 |
| **Melanogenesis** | 0.00217060694801 | 24 | 3 |
| **Pathways in cancer** | 0.00261106413774 | 64 | 3 |
| **Phosphatidylinositol signaling system** | 0.00306706480574 | 17 | 3 |
| **GABAergic synapse** | 0.00812168204787 | 17 | 3 |
| **Cholinergic synapse** | 0.0135886290579 | 24 | 3 |
| **Thyroid hormone signaling pathway** | 0.0135886290579 | 24 | 3 |
| **Oxytocin signaling pathway** | 0.0135886290579 | 31 | 3 |
| **Axon guidance** | 0.0151165892452 | 22 | 3 |
| **ErbB signaling pathway** | 0.0177303979755 | 20 | 3 |
| **Long-term depression** | 0.0177303979755 | 13 | 3 |
| **Dorso-ventral axis formation** | 0.0264846797459 | 9 | 3 |
| **Estrogen signaling pathway** | 0.0279078564569 | 18 | 3 |
| **Wnt signaling pathway** | 0.0319560215051 | 25 | 3 |
| **Proteoglycans in cancer** | 0.0341279346781 | 38 | 3 |
| **MAPK signaling pathway** | 0.0353157062224 | 41 | 3 |

**Supplemental Table 8.** Predicted pathway interactions of significantly up-regulated microRNAs in middle-aged individuals (50-80 years of age) compared to nonagenarians. Pathway interactions with p-value < 0.05 were derived using DIANA Tools (Vlachos et al., 2015).

**Supplemental Table 9.** Differentially expressed miRNAs in young individuals (<40 years of age) compared to middle-aged individuals (50-80 years of age). logFC indicates log fold change and logCPM represents log counts per million indicative of expression levels; p-value < 0.05 was considered significant.

|  | **logFC** | **logCPM** | **p-value** |
| --- | --- | --- | --- |
| **hsa-miR-4732-3p** | -3.2407505 | 5.38264618 | 0.0203243 |
| **hsa-miR-93-3p** | -3.0869260 | 4.60663791 | 0.0152597 |
| **hsa-let-7i-3p** | -2.7368425 | 4.70084674 | 0.0268640 |
| **hsa-miR-487a-5p** | -2.5378338 | 4.49140078 | 0.0329253 |
| **hsa-miR-1261** | -2.3866470 | 4.40926818 | 0.0443637 |
| **hsa-miR-1296-5p** | -2.3600904 | 5.31207638 | 0.0176923 |
| **hsa-miR-324-5p** | -1.8805457 | 5.24149202 | 0.0352703 |
| **hsa-miR-451a** | -1.4294513 | 16.1951268 | 0.0292556 |
| **hsa-miR-584-5p** | 1.0684308 | 11.6084869 | 0.0078417 |
| **hsa-miR-125a-5p** | 1.0787864 | 10.7545324 | 0.0297724 |
| **hsa-miR-199b-3p** | 1.1646391 | 11.4674282 | 0.0225802 |
| **hsa-miR-340-5p** | 1.2707677 | 8.63434012 | 0.0386588 |
| **hsa-miR-26a-5p** | 1.2725177 | 15.6034263 | 0.0093806 |
| **hsa-miR-199a-3p** | 1.3696738 | 11.4711473 | 0.0081505 |
| **hsa-miR-155-5p** | 1.6862887 | 8.95560767 | 0.0156742 |
| **hsa-let-7d-5p** | 1.6920893 | 12.9738411 | 0.0382247 |
| **hsa-miR-10a-5p** | 1.8027767 | 9.22539916 | 0.0086741 |
| **hsa-miR-126-5p** | 1.8386699 | 9.31939955 | 0.0204423 |
| **hsa-miR-200c-3p** | 1.9096697 | 7.14353797 | 0.0215203 |
| **hsa-let-7e-5p** | 1.9489281 | 8.88407826 | 0.0309626 |
| **hsa-miR-126-3p** | 2.0798008 | 14.3319141 | 0.0015016 |
| **hsa-miR-122-5p** | 2.1047310 | 9.90241413 | 0.0429913 |
| **hsa-miR-493-5p** | 2.2196616 | 8.7941083 | 0.0215798 |
| **hsa-miR-550a-5p** | 2.6747094 | 6.08959382 | 0.0166173 |
| **hsa-miR-340-3p** | 2.7671663 | 6.9697121 | 0.0390388 |
| **hsa-miR-203a-3p** | 2.9943662 | 4.84006749 | 0.0445934 |
| **hsa-miR-1-3p** | 3.1671729 | 9.12707332 | 0.0027009 |
| **hsa-miR-411-5p** | 4.1491262 | 7.17875399 | 0.0217092 |

**Supplemental Table 10.** Predicted pathway interactions of significantly down-regulated microRNAs in young individuals (<40 years of age) compared to middle-aged individuals (50-80 years of age ~~of age~~). Pathway interactions with p-value < 0.05 were derived using DIANA Tools (Vlachos et al., 2015).

| **KEGG pathway** | **p-value** | **#genes** | **#miRNAs** |
| --- | --- | --- | --- |
| **Amphetamine addiction** | 8.28048005771E-06 | 13 | 6 |
| **Circadian entrainment** | 0.000161082768299 | 15 | 5 |
| **Signaling pathways regulating pluripotency of stem cells** | 0.00170835663592 | 20 | 6 |
| **Proteoglycans in cancer** | 0.00471583789359 | 22 | 7 |
| **TGF-beta signaling pathway** | 0.00548997584217 | 9 | 5 |
| **Long-term potentiation** | 0.00548997584217 | 13 | 6 |
| **Oxytocin signaling pathway** | 0.00664019184221 | 22 | 6 |
| **Estrogen signaling pathway** | 0.00820530253824 | 11 | 6 |
| **Glioma** | 0.0153917267229 | 9 | 5 |
| **Hedgehog signaling pathway** | 0.0218188478318 | 9 | 4 |
| **Thyroid hormone signaling pathway** | 0.0218188478318 | 16 | 6 |
| **Cholinergic synapse** | 0.027578617528 | 15 | 6 |
| **Dopaminergic synapse** | 0.0358918880813 | 18 | 6 |
| **Endocrine and other factor-regulated calcium reabsorption** | 0.0363575772625 | 7 | 4 |
| **Long-term depression** | 0.041431715061 | 10 | 5 |
| **Gap junction** | 0.0426154739648 | 13 | 5 |
| **cGMP-PKG signaling pathway** | 0.0448784148923 | 19 | 6 |

**Supplemental Table 11.** Predicted pathway interactions of significantly up-regulated microRNAs in young individuals (<40 years of age) compared to middle-aged individuals (50-80 years of age). Pathway interactions with p-value < 0.05 were derived using DIANA Tools (Vlachos et al., 2015).

| **KEGG pathway** | **p-value** | **#genes** | **#miRNAs** |
| --- | --- | --- | --- |
| **Mucin type O-Glycan biosynthesis** | 1.31380459825E-11 | 16 | 14 |
| **Hippo signaling pathway** | 1.99156534784E-06 | 72 | 18 |
| **Proteoglycans in cancer** | 1.99156534784E-06 | 98 | 18 |
| **Transcriptional misregulation in cancer** | 1.17635362048E-05 | 86 | 17 |
| **FoxO signaling pathway** | 0.000142160210664 | 71 | 19 |
| **Ras signaling pathway** | 0.000181849356995 | 111 | 19 |
| **Neurotrophin signaling pathway** | 0.000196282786091 | 64 | 19 |
| **TGF-beta signaling pathway** | 0.000341796812879 | 44 | 18 |
| **Axon guidance** | 0.000341796812879 | 63 | 19 |
| **Pathways in cancer** | 0.000835330437441 | 180 | 19 |
| **PI3K-Akt signaling pathway** | 0.000865770520216 | 154 | 19 |
| **Thyroid hormone signaling pathway** | 0.0010781688584 | 56 | 16 |
| **Small cell lung cancer** | 0.00114818366024 | 47 | 16 |
| **Wnt signaling pathway** | 0.00114818366024 | 69 | 17 |
| **Adrenergic signaling in cardiomyocytes** | 0.00114818366024 | 68 | 18 |
| **Viral carcinogenesis** | 0.00222472350689 | 80 | 15 |
| **Chronic myeloid leukemia** | 0.00274741144865 | 38 | 16 |
| **GABAergic synapse** | 0.00274741144865 | 43 | 18 |
| **Signaling pathways regulating pluripotency of stem cells** | 0.00274741144865 | 69 | 19 |
| **MAPK signaling pathway** | 0.00335085974651 | 117 | 18 |
| **Morphine addiction** | 0.00335085974651 | 44 | 18 |
| **cAMP signaling pathway** | 0.00465182583603 | 92 | 19 |
| **T cell receptor signaling pathway** | 0.00516435545652 | 53 | 16 |
| **Hepatitis B** | 0.00516435545652 | 61 | 18 |
| **Focal adhesion** | 0.00516435545652 | 95 | 19 |
| **mTOR signaling pathway** | 0.00773704266028 | 33 | 17 |
| **Glioma** | 0.00773704266028 | 31 | 17 |
| **Glutamatergic synapse** | 0.00773704266028 | 54 | 18 |
| **ECM-receptor interaction** | 0.00855241153129 | 37 | 17 |
| **ErbB signaling pathway** | 0.0118368865565 | 44 | 17 |
| **Rap1 signaling pathway** | 0.0138106330505 | 94 | 19 |
| **N-Glycan biosynthesis** | 0.0157504760183 | 21 | 17 |
| **Long-term potentiation** | 0.0172018135652 | 35 | 18 |
| **Adipocytokine signaling pathway** | 0.0200836021643 | 35 | 18 |
| **Glycosaminoglycan biosynthesis - heparan sulfate / heparin** | 0.0213337695524 | 14 | 7 |
| **Renal cell carcinoma** | 0.0213337695524 | 34 | 16 |
| **Regulation of actin cytoskeleton** | 0.0213337695524 | 92 | 19 |
| **cGMP-PKG signaling pathway** | 0.0241455239688 | 74 | 19 |
| **Protein processing in endoplasmic reticulum** | 0.028954740957 | 74 | 19 |
| **Type II diabetes mellitus** | 0.0311545521624 | 24 | 16 |
| **Retrograde endocannabinoid signaling** | 0.0340500935562 | 48 | 18 |
| **Arrhythmogenic right ventricular cardiomyopathy (ARVC)** | 0.0474450920102 | 36 | 18 |
| **Insulin signaling pathway** | 0.0490852069319 | 63 | 19 |

**Supplemental Table 12.** Predicted pathway interactions of significantly down-regulated microRNAs demonstrating genotype associations in young, middle-aged, and nonagenarian individuals (<40, 50-80, and =≥ 90 years of age, respectively). Pathway interactions with p-value < 0.05 were derived using DIANA Tools (Vlachos et al., 2015).

| **KEGG pathway** | **p-value** | **#genes** | **#miRNAs** |
| --- | --- | --- | --- |
| **TGF-beta signaling pathway** | 1.43240678258E-08 | 33 | 7 |
| **Signaling pathways regulating pluripotency of stem cells** | 2.90749077479E-06 | 48 | 7 |
| **FoxO signaling pathway** | 0.000109732189652 | 45 | 7 |
| **Mucin type O-Glycan biosynthesis** | 0.000209115824587 | 8 | 4 |
| **Hippo signaling pathway** | 0.000342491806834 | 50 | 8 |
| **Glycosaminoglycan biosynthesis - chondroitin sulfate / dermatan sulfate** | 0.000530289444272 | 7 | 4 |
| **Pathways in cancer** | 0.000540687974547 | 100 | 8 |
| **Morphine addiction** | 0.00144935193108 | 28 | 6 |
| **Melanoma** | 0.00161549619296 | 26 | 6 |
| **Proteoglycans in cancer** | 0.00252218765337 | 55 | 7 |
| **GABAergic synapse** | 0.00268366188344 | 23 | 6 |
| **Biotin metabolism** | 0.0028675662271 | 1 | 1 |
| **Long-term potentiation** | 0.00424001399624 | 25 | 6 |
| **Renal cell carcinoma** | 0.00496141403964 | 23 | 7 |
| **Glutamatergic synapse** | 0.0071234466384 | 31 | 6 |
| **ErbB signaling pathway** | 0.0071234466384 | 27 | 7 |
| **Gap junction** | 0.0078695329794 | 24 | 8 |
| **Axon guidance** | 0.00794565928776 | 38 | 8 |
| **Sphingolipid metabolism** | 0.00809945330074 | 15 | 5 |
| **Non-small cell lung cancer** | 0.0144211269693 | 17 | 4 |
| **Nicotine addiction** | 0.0144211269693 | 13 | 5 |
| **Maturity onset diabetes of the young** | 0.0208156169487 | 9 | 3 |
| **Glioma** | 0.0273326123028 | 19 | 7 |
| **Rap1 signaling pathway** | 0.0273326123028 | 54 | 8 |
| **Thyroid cancer** | 0.0283922056099 | 9 | 4 |
| **Estrogen signaling pathway** | 0.0334530193557 | 23 | 7 |
| **Prolactin signaling pathway** | 0.0383245366802 | 19 | 5 |
| **cGMP-PKG signaling pathway** | 0.0403508834873 | 43 | 8 |
| **Ubiquitin mediated proteolysis** | 0.0403508834873 | 38 | 8 |
| **Chronic myeloid leukemia** | 0.0411386398507 | 22 | 6 |
| **Melanogenesis** | 0.0416690724017 | 29 | 8 |
| **Hedgehog signaling pathway** | 0.0424263082685 | 17 | 6 |
| **p53 signaling pathway** | 0.0474267456519 | 21 | 6 |

**Supplemental Table 13.** Predicted pathway interactions of significantly up-regulated microRNAs demonstrating genotype associations in young, middle-aged, and nonagenarian individuals (<40, 50-80, and =≥ 90 years of age, respectively). Pathway interactions with p-value < 0.05 were derived using DIANA Tools (Vlachos et al., 2015). 

| **KEGG pathway** | **p-value** | **#genes** | **#miRNAs** |
| --- | --- | --- | --- |
| **Axon guidance** | 3.44725335832E-07 | 52 | 12 |
| **Proteoglycans in cancer** | 3.59768275239E-07 | 81 | 13 |
| **Estrogen signaling pathway** | 3.59768275239E-07 | 45 | 14 |
| **Long-term potentiation** | 4.94728894709E-05 | 36 | 13 |
| **Melanogenesis** | 5.0119868357E-05 | 46 | 13 |
| **Glioma** | 0.000222908368341 | 29 | 12 |
| **Morphine addiction** | 0.000222908368341 | 36 | 13 |
| **ErbB signaling pathway** | 0.000330138576082 | 38 | 12 |
| **Amphetamine addiction** | 0.00060964742317 | 29 | 11 |
| **Cholinergic synapse** | 0.00060964742317 | 49 | 12 |
| **Regulation of actin cytoskeleton** | 0.00060964742317 | 80 | 12 |
| **Endometrial cancer** | 0.00060964742317 | 26 | 12 |
| **Arrhythmogenic right ventricular cardiomyopathy (ARVC)** | 0.000704857610903 | 30 | 10 |
| **Glutamatergic synapse** | 0.000991095030529 | 45 | 13 |
| **Signaling pathways regulating pluripotency of stem cells** | 0.00116539324443 | 53 | 12 |
| **Prostate cancer** | 0.00204544740349 | 38 | 12 |
| **Adrenergic signaling in cardiomyocytes** | 0.00204544740349 | 57 | 13 |
| **Endocytosis** | 0.00204544740349 | 73 | 14 |
| **Gap junction** | 0.00233065226878 | 34 | 12 |
| **Transcriptional misregulation in cancer** | 0.00233065226878 | 58 | 13 |
| **Nicotine addiction** | 0.00278698233303 | 16 | 10 |
| **Thyroid hormone signaling pathway** | 0.00278698233303 | 45 | 11 |
| **Focal adhesion** | 0.00278698233303 | 76 | 13 |
| **Pathways in cancer** | 0.00278698233303 | 122 | 13 |
| **Renal cell carcinoma** | 0.00416489258402 | 31 | 11 |
| **Circadian rhythm** | 0.00505758499886 | 16 | 11 |
| **Circadian entrainment** | 0.00741771929982 | 43 | 13 |
| **cGMP-PKG signaling pathway** | 0.00768121912585 | 60 | 13 |
| **Ras signaling pathway** | 0.00861399975555 | 75 | 14 |
| **Oxytocin signaling pathway** | 0.00888737212704 | 58 | 14 |
| **Non-small cell lung cancer** | 0.00991695034464 | 24 | 11 |
| **Hippo signaling pathway** | 0.0113033257358 | 50 | 11 |
| **Fc gamma R-mediated phagocytosis** | 0.0113033257358 | 37 | 11 |
| **Wnt signaling pathway** | 0.0114674278731 | 47 | 12 |
| **Retrograde endocannabinoid signaling** | 0.0139544505496 | 39 | 13 |
| **Dopaminergic synapse** | 0.0162041386794 | 51 | 14 |
| **Long-term depression** | 0.018185591107 | 24 | 12 |
| **Dorso-ventral axis formation** | 0.0213285244869 | 14 | 8 |
| **GABAergic synapse** | 0.030655877598 | 33 | 11 |
| **Rap1 signaling pathway** | 0.0331220735993 | 67 | 14 |
| **Colorectal cancer** | 0.0347412522234 | 24 | 10 |
| **Adherens junction** | 0.036795878147 | 25 | 11 |
| **FoxO signaling pathway** | 0.0396434524428 | 50 | 10 |
| **Cocaine addiction** | 0.0396434524428 | 19 | 10 |
| **AMPK signaling pathway** | 0.0396434524428 | 43 | 11 |
| **GnRH signaling pathway** | 0.0396434524428 | 34 | 13 |
| **mTOR signaling pathway** | 0.0425251540304 | 25 | 8 |
| **Chronic myeloid leukemia** | 0.0427923537345 | 27 | 12 |
| **cAMP signaling pathway** | 0.0438262644328 | 67 | 13 |
| **Salivary secretion** | 0.0442563544192 | 33 | 13 |
| **Bacterial invasion of epithelial cells** | 0.0468392821254 | 26 | 11 |
| **Neurotrophin signaling pathway** | 0.0468392821254 | 44 | 12 |
| **Inflammatory mediator regulation of TRP channels** | 0.0468392821254 | 36 | 13 |
| **HIF-1 signaling pathway** | 0.0468392821254 | 37 | 14 |
| **Phosphatidylinositol signaling system** | 0.0468392821254 | 31 | 14 |


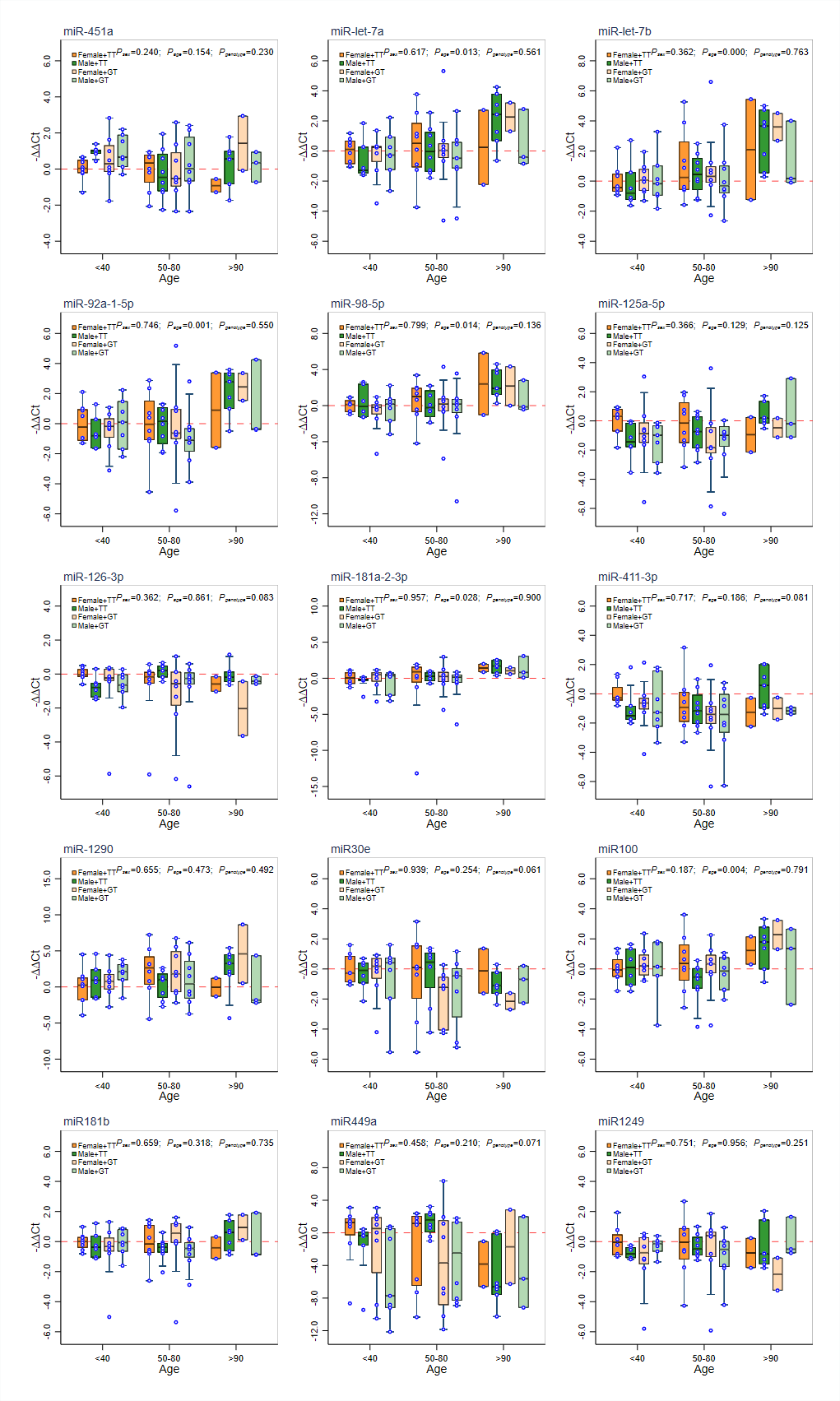
**Supplemental Figure 2.** Boxplots demonstrating expression of 15 miRNAs quantified through RT-qPCR and their association with sex, age and genotype. The expression of the 15 miRNAs were measured as
-∆∆Ct normalized using the housekeeping miRNA miR-16 and TT females of <40 years old as a baseline subgroup. Multi-factor ANOVA analysis was performed to test the partial effect of sex, age and genotype on the expression of each miRNA. The results suggest that the expressions of miR-let-7a, miR-let-7b, miR-92a-1-5p, miR-98-5p, miR-181a-2-3p, and miR100 are significantly up-regulated in people of =≥ 90 years old (p=0.013, p<0.0001, p=0.001, p=0.014, p=0.028 and p=0.004 respectively), and that the expression of the 15 studied miRNAs are not significantly associated with sex and genotype.


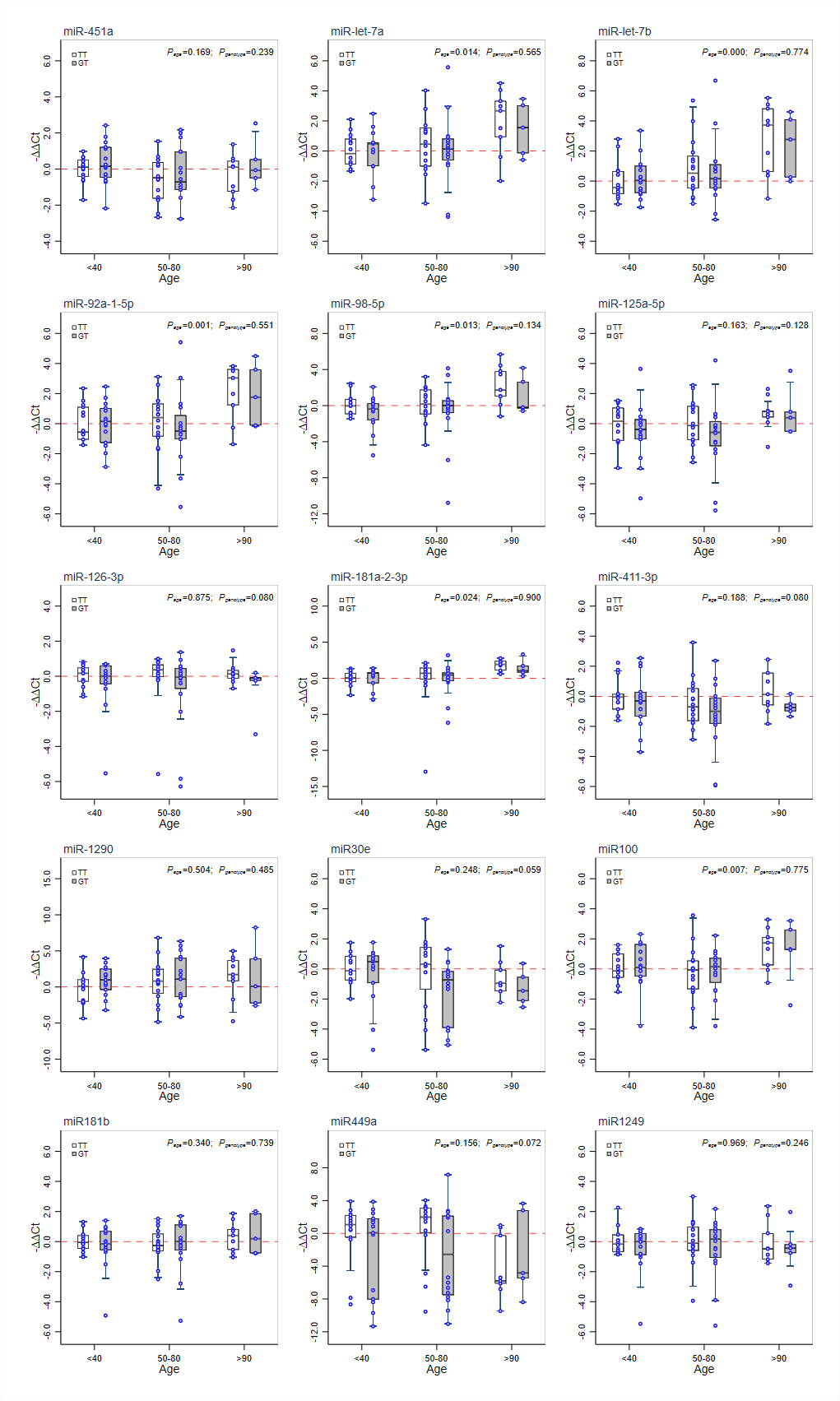


**Supplemental Figure 3.** Boxplots showing expression of 15 miRNAs and its association with age and genotype after excluding sex. The expression of the 15 miRNAs was measured as -∆∆Ct normalized using miR16 as a house-keeping miRNA and TT individuals of <40 years old as a baseline subgroup. Multi-factor ANOVA analysis was performed to test the partial effect of age and genotype on the expression of each miRNA. The results suggest that the expressions of miR-let-7a, miR-let-7b, miR-92a-1-5p, miR-98-5p, miR-181a-2-3p, and miR100 are significantly up-regulated in people of =≥ 90 years old (p=0.014, p<0.001, p=0.001, p=0.013, p=0.024 and p=0.007 respectively), and that the expressions of the 15 studied miRNAs are not significantly associated with genotype.


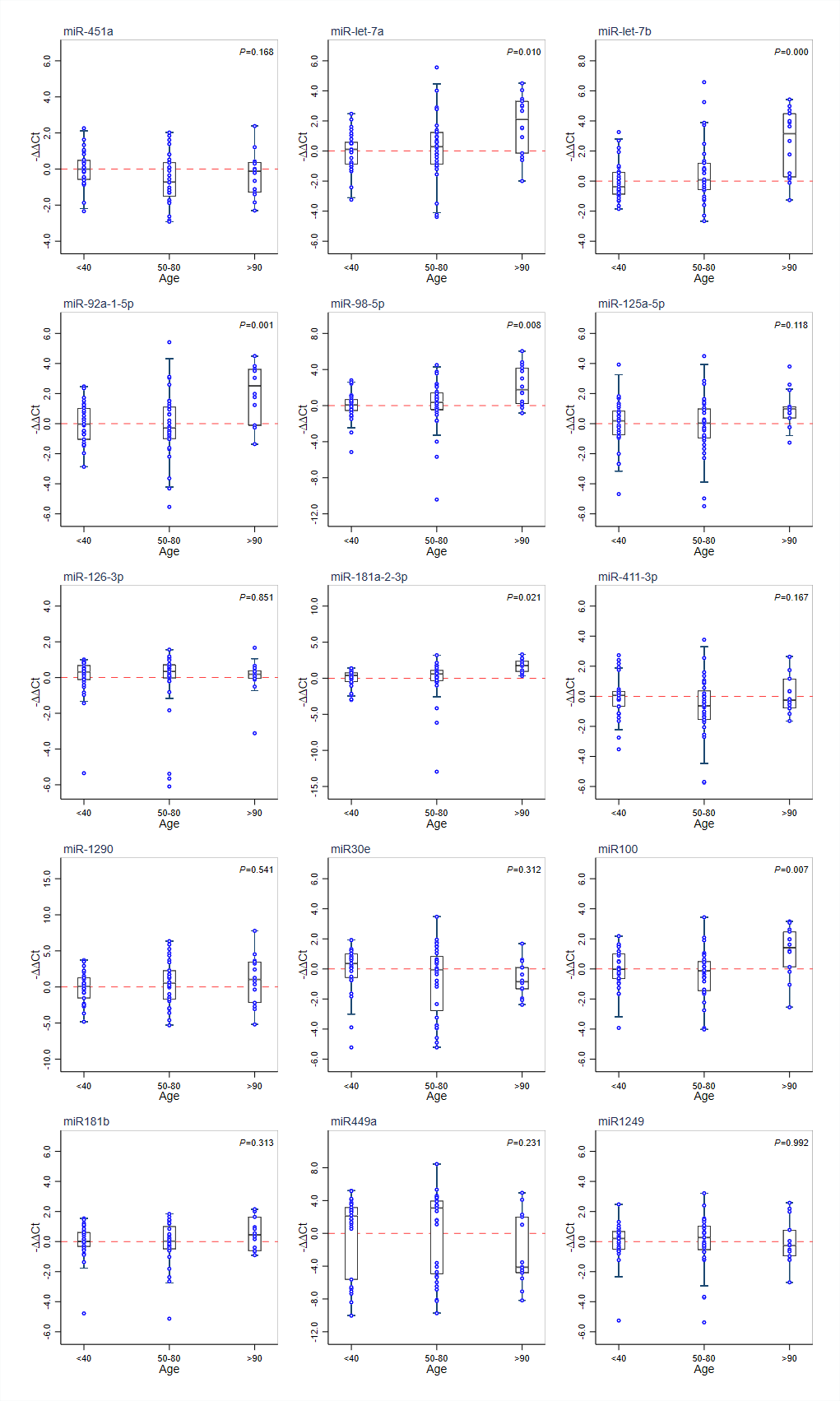
**Supplemental Figure 4**. Boxplots demonstrating expression of 15 miRNAs and its association with age after excluding sex and genotype. The expression of the 15 miRNAs was measured as -∆∆Ct normalized using miR16 as a house-keeping miRNA and the group <40 years old as a baseline subgroup. One-way ANOVA analysis was performed to test the partial effect of age on the expression of each miRNA. The results suggest that the expressions of miR-let-7a, miR-let-7b, miR-92a-1-5p, miR-98-5p, miR-181a-2-3p, and miR100 are significantly up-regulated in people of =≥ 90 years old (p=0.010, p<0.0001, p=0.001, p=0.008, p=0.021 and p=0.007 respectively).


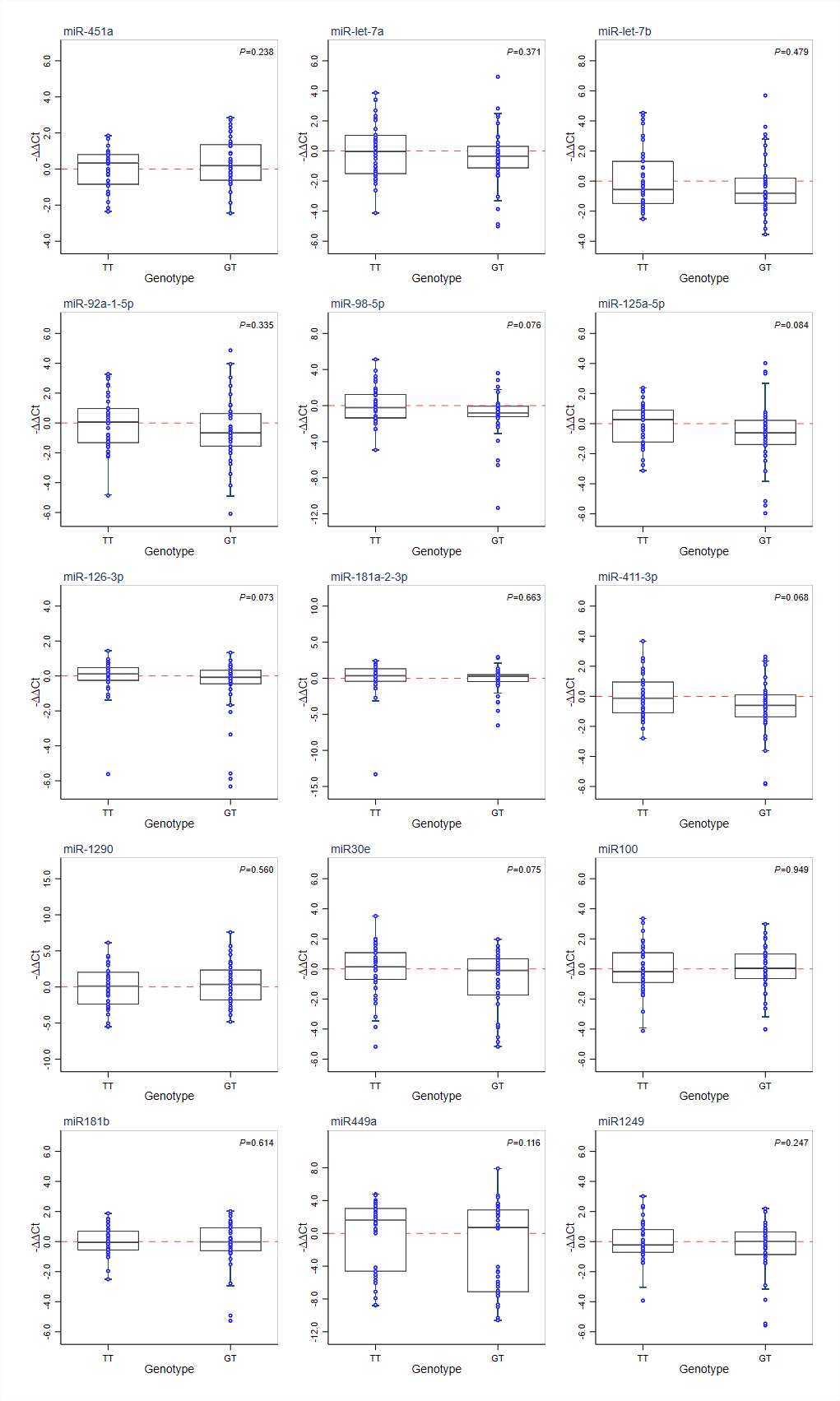


**Supplemental Figure 5.** Boxplots showing expression of 15 miRNAs and its association with genotype after excluding sex and age. The expression of the 15 miRNAs was measured as -∆∆Ct normalized using miR16 as a house-keeping miRNA and the TT genotype as a baseline subgroup. One-way ANOVA analysis was performed to test the effect of genotype on the expression of each miRNA. The results suggest that the expressions of the 15 studied miRNAs are not significantly different between the two genotypes.

**
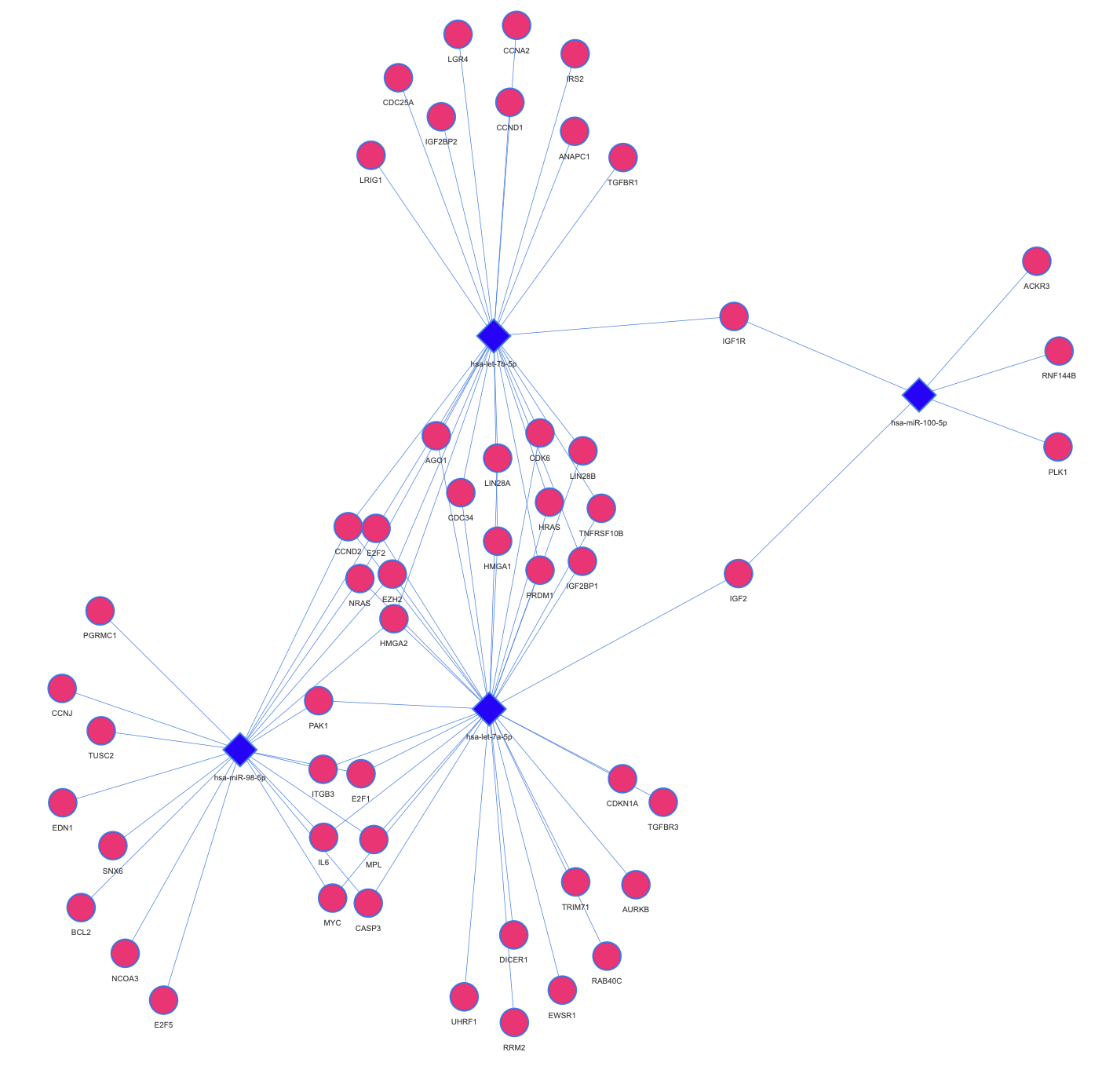
Supplemental Figure 6.** miRNA-gene interactions. Generated using the miRNA-target enrichment and network-based analysis tool MIENTURNET (Licursi et al. 2019).
